# A bibliometric review of research on resilience, motivation and prisoners, 1912-2024

**DOI:** 10.12688/f1000research.164019.2

**Published:** 2025-10-22

**Authors:** Zalmizy Hussin, Md Zawawi Abu Bakar, Mohd Ahsani A.Malek, NoorSuzana Mohd Shariff, Siti Rohana Ahmad

**Affiliations:** 1 School of Applied Psychology, Social Work and Policy, Universiti Utara Malaysia, Sintok, Kedah, Malaysia; 2 Department of Community Health, Advanced Medical and Dental Institute, Universiti Sains Malaysia, Kepala Batas, Pulau Pinang, Malaysia; 3 Family Health Department, Kedah State Health Department, Alor Setar, Malaysia

**Keywords:** Resilience, motivation, prisoners, rehabilitation, bibliometric analysis

## Abstract

**Background:**

Research on resilience and motivation among incarcerated individuals has gained increasing academic interest due to its relevance in rehabilitation, reducing recidivism, and promoting societal reintegration. Prisoners often face psychological distress, social isolation, and behavioral challenges, making resilience and motivation essential for enduring incarceration and engaging in rehabilitative efforts. However, a comprehensive bibliometric analysis that maps the evolution, influence, and thematic development of this research area remains limited.

**Methods:**

This study aims to examine publication trends, key contributors, major thematic domains, and intellectual structures in resilience and motivation research related to prisoners from 1912 to 2024. Using Scopus-indexed literature, a bibliometric analysis was conducted employing co-authorship networks, keyword co-occurrence, and citation analysis to assess research output, collaboration patterns, and thematic progression.

**Results:**

Findings reveal a significant surge in research since the early 2000s, with the United States, United Kingdom, and Germany leading in publication volume. Keyword analysis identifies four dominant thematic clusters: psychological resilience and coping, addiction rehabilitation, post-incarceration reintegration strategies, and decision-making within correctional environments. Citation analysis highlights pivotal works that have shaped the field, reflecting a shift from punitive correctional models toward rehabilitative, psychological, and policy-oriented approaches. Despite advancements, gaps remain in cross-cultural perspectives, gender-specific interventions, and post-release support systems.

**Conclusions:**

The findings underscore the need for global collaboration, interdisciplinary approaches, and innovative rehabilitation strategies to advance resilience and motivation research in correctional contexts. This review contributes to the development of evidence-based policies, correctional education, and rehabilitation frameworks aimed at improving prisoner well-being, reducing recidivism, and fostering successful reintegration into society.

## 1. Introduction

### 1.1 Research background

Research on resilience and motivation holds significant importance across disciplines such as psychology, sociology, criminology, and public health ( Adams, 2022; Schulz et al., 2023). Resilience—the dynamic process of adapting and recovering from adversity, stress, or major obstacles—is widely recognized as a key determinant of psychological well-being ( Adams, 2022; Tomaszewski, 2023; Uddin et al., 2012). Motivation, whether intrinsic or extrinsic, drives individuals to pursue goals and change behavior ( Schulz et al., 2023; Deci & Ryan, 2012). These two constructs often intersect in contexts characterized by chronic stress and restricted autonomy, where recovery and personal growth are essential for survival and development, particularly in criminogenic or institutional environments ( Passas, 2024).

Incarcerated individuals embody this intersection. Life in prison is frequently associated with the loss of freedom, stigma, social disconnection, and exposure to violence ( Curley et al., 2025). These stressors often compound pre-existing vulnerabilities such as mental illness or substance dependency, making rehabilitation and reintegration especially challenging ( Batistich et al., 2025; Klinoff et al., 2018). Building psychological resilience and strengthening motivation is therefore crucial for inmates to engage in constructive activities such as therapy, education, and vocational training ( Paquette et al., 2025).

Over the last century, correctional systems worldwide have gradually shifted from punitive models to rehabilitative frameworks that emphasize psychological and social support ( Ahmed et al., 2025; Durkin, 2025). This paradigm shift has fueled academic interest in how prison environments can foster resilience and motivation to reduce recidivism and improve post-release reintegration ( Dewey et al., 2024). Scholars have increasingly investigated how resilience and motivation can support personal, psychological, and behavioral development among incarcerated individuals ( Powrie, 2025).

### 1.2 Review of literature

In developmental psychology, early research on resilience focused on how children overcame adversity related to poverty, abuse, or neglect ( David & Towl, 2021). Foundational studies by Werner and Smith in the 1950s and 1980s emphasized traits such as emotional regulation, problem-solving skills, and the presence of supportive adult relationships ( Whitehead et al., 2014). These characteristics enabled individuals to navigate hardship, supporting the notion that resilience is both complex and dynamic ( Pamungkas et al., 2023). In modern scholarships, resilience research now includes family, community, and structural dimensions.

In correctional settings, resilience is essential for managing psychological stressors such as confinement, loss of autonomy, and institutional aggression. Evidence shows that cognitive strategies, peer support, and rehabilitation programs can significantly enhance resilience ( Carmo et al., 2024). Mindfulness and cognitive behavioral therapy (CBT) have been found to improve emotional regulation and stress management among inmates ( Derlic, 2022; Harding-White et al., 2024). Peer-led support programs have also demonstrated value in fostering resilience and promoting pro-social behavior ( Riley et al., 2019).

Motivation studies have evolved alongside resilience research. Influential theories such as Maslow’s hierarchy of needs and Bandura’s self-efficacy model highlight how fulfilling psychological needs and fostering agency contribute to behavioral change ( Pastwa-Wojciechowska & Guzińska, 2024; Shield, 2025). More recent work, particularly Deci and Ryan’s Self-Determination Theory (SDT), differentiates between intrinsic and extrinsic motivation ( Ntsame Sima et al., 2024). Intrinsic motivation—driven by personal values and interest—tends to sustain long-term behavioral change more effectively than extrinsic incentives ( Moller et al., 2014; Ryan & Reeve, 2021).

Incarcerated individuals require significant motivation to engage in rehabilitative programs. Approaches like motivational interviewing have been shown to enhance participation in education and occupational training. Goal-setting initiatives, in which prisoners develop attainable and meaningful goals, are also associated with improved behavioral outcomes. Notably, resilience is linked to higher levels of self-efficacy and determination, both of which are essential for success during and after incarceration ( Pandya, 2022).

Multidisciplinary research increasingly demonstrates that resilience and motivation significantly influence psychological well-being and rehabilitation outcomes among prisoners. Interventions aimed at strengthening resilience can also enhance motivation by promoting autonomy, competence, and relatedness—central constructs of self-determination theory ( Huang et al., 2020). Conversely, boosting motivation may improve resilience by fostering confidence in one’s ability to overcome challenges ( Navickienė & Vasiliauskas, 2024; Schulz et al., 2023).

Despite this progress, significant gaps remain. Much of the literature on resilience and motivation in prison settings is Western-centric, limiting its generalizability across diverse cultural and socio-economic environments. Further research is needed to understand how cultural norms, systemic inequalities, and resource constraints shape resilience and motivation within global correctional systems. Additionally, the application of digital technologies—such as virtual reality, mobile apps, and artificial intelligence—for supporting inmate rehabilitation is promising yet underexplored.

Finally, most research to date has focused on individual-level interventions, overlooking broader systemic and structural influences. Examining how prison policies, institutional environments, and staff-prisoner interactions affect psychological outcomes could lead to more supportive correctional practices. Moreover, limited attention has been given to the role of resilience and motivation in the post-release phase. Research on how these constructions influence employment, mental health, and community reintegration is essential for designing effective recidivism-reduction strategies.

### 1.3 Study objectives

This bibliometric review aims to analyze trends, methodologies, and thematic developments in research on resilience and motivation among incarcerated individuals between 1912 and 2024. The specific objectives are to:

1) Track the historical development of scholarly work on resilience and motivation in correctional contexts, including major milestones and future directions.
2) Identify and evaluate leading countries, institutions, and scholars contributing to this field, as well as their patterns of collaboration and influence.
3) Analyze dominant research themes, frequently used keywords, and citation patterns to guide future research efforts.

This review integrates fragmented literature to map the intellectual evolution of this field and inform the development of evidence-based interventions and correctional policies aimed at improving mental health, reducing recidivism, and promoting the successful reintegration of prisoners into society.

## 2. Materials and Methods

### 2.1 Source identification

This study utilized the Scopus database as the primary source of bibliographic data due to its comprehensive coverage in the social sciences, psychology, and public health disciplines. Compared to alternatives like Web of Science, Scopus offers broader indexing and improved accessibility to high-quality scholarly publications ( Pranckutė, 2021; Tennant, 2020). The review included a diverse range of documents, including journal articles, books, book chapters, and conference proceedings. To ensure inclusivity, the search strategy used the keywords: “resilience” OR “motivation” AND “prisoners.” No start year was specified in the search query, allowing for the inclusion of the earliest available records.

The study adhered to the PRISMA (Preferred Reporting Items for Systematic Reviews and Meta-Analyses) guidelines to ensure transparency in the identification, screening, and selection of documents ( Moher et al., 2010; Pham & Le, 2024). An initial Scopus search yielded 1,309 documents. After removing two duplicates, 1,307 records remained for screening. A title and abstract review excluded 50 documents unrelated to prisoner resilience or motivation. A further 20 records were excluded during full-text assessment for lacking methodological relevance. This process resulted in the inclusion of 1,227 articles for qualitative synthesis, and 10 for quantitative synthesis (meta-analysis).

### 2.2 Data analysis

The final dataset comprised 1,309 Scopus-indexed publications focusing on *prisoner resilience* and *motivation* from 1912 to 2024. Extracted bibliographic elements included authorship, publication year, source title, institutional affiliation, country, keywords, and citation metrics. Both descriptive and bibliometric techniques were applied to identify trends, collaboration patterns, and structural relationships within the field.

Descriptive statistics were first employed to examine the temporal distribution of publications, highlighting distinct periods of scholarly growth and thematic diversification. Institutional and country-level affiliations were analyzed to identify leading contributors and regions of research concentration. Citation and co-citation analyses were then used to determine the intellectual structure of the field, pinpointing influential scholars and foundational works ( Ding et al., 2014; Robledo-Giraldo et al., 2023).

Bibliometric mapping and visualization were conducted using VOSviewer software (version 1.6.19) developed by Van Eck and Waltman. The full dataset was exported from Scopus in *CSV format* with complete bibliographic information. To ensure transparency and reproducibility, a multi-stage analytical protocol was implemented as follows:

1. Data Cleaning: Duplicate records, incomplete metadata, non-English titles, errata, and editorial materials were removed manually using Microsoft Excel to ensure data consistency and quality control.
2. Keyword Standardization: Synonymous or variant keywords were consolidated to avoid redundancy (e.g., *resilience* and *resiliency*; *motivation* and *motivational factors*; *inmates* and *prisoners*). The *thesaurus* function in VOSviewer was utilized to merge similar terms and unify terminology across the dataset.
3. Software Configuration: Analyses were performed using standardized parameters to enhance comparability:
  ○ *Minimum number of keyword occurrences:* 5
  ○ *Resolution:* 0.85
  ○ *Normalization method:* Association Strength
  ○ *Visualization types:* Network, Overlay, and Density maps
4. Thematic and Temporal Mapping: Co-occurrence and co-authorship analyses were performed separately for keywords, authors, and institutions. The overlay visualization function was used to trace the temporal evolution of major research themes, applying a year-based gradient color scheme to visualize how topics have shifted from early studies on coping and adjustment to contemporary emphases on rehabilitation, transformation, and reintegration.
5. Reliability and Reproducibility: All data-handling steps, search queries, and software settings were archived as a supplementary file (see *Data Availability Statement*) to enable replication and extension by future researchers.

The integration of these analytical approaches provided a comprehensive evaluation of the field’s development, capturing both structural and temporal dimensions of scholarly productivity. This combination of descriptive and network-based techniques enabled the identification of dominant thematic clusters, emerging interdisciplinary linkages, and geographic disparities in research output—thereby fulfilling the study’s objectives of mapping intellectual influence, collaboration, and thematic evolution ( Lim et al., 2024).

This methodological triangulation not only ensures analytical robustness but also situates bibliometric patterns within broader theoretical interpretations of resilience and motivation in correctional contexts.

### 2.3 Inclusion and exclusion criteria

Rigorous inclusion and exclusion criteria were established to ensure the quality and relevance of the selected documents. Eligible publications included empirical studies, theoretical articles, reviews, book chapters, and conference proceedings with a substantive focus on resilience or motivation among prisoners.

Only English-language documents were included to maintain linguistic consistency and maximize accessibility to an international audience. The time span extended from 1912 to 2024, enabling both historical and contemporary trends to be captured. Relevance was determined through title and abstract screening.

Documents were excluded if they lacked empirical or conceptual depth, such as editorial notes, brief reports, letters to the editor, and retracted publications. Non-English materials were excluded for consistency. Publications without identifiable titles or author information were also removed to maintain dataset integrity.

These selection criteria ensured the inclusion of high-quality, thematically relevant literature and enhanced the methodological rigor of the bibliometric analysis.

### 2.4 Reliability

The study ensured reliability through standardized procedures and adherence to established bibliometric protocols ( Babu & Kohli, 2023). The selection of Scopus as the data source enhanced data validity, given its peer-reviewed and indexed content.

The use of clearly defined inclusion and exclusion criteria minimized selection bias and enhanced reproducibility. Compliance with PRISMA guidelines ensured transparency in the review process, from initial identification to final synthesis.

VOSviewer software provided a consistent and replicable platform for network and trend analysis, including co-authorship, co-citation, and keyword mapping. Duplicate and incomplete records were eliminated, and only verified entries were retained in the final dataset.

These methodological safeguards contributed to the robustness, consistency, and credibility of the study’s findings, offering a dependable foundation for future research in this field.

## 3. Results and Discussion

### 3.1 Year of publication

Table 1 presents the distribution of publications on resilience and motivation among prisoners from 1912 to 2025. Over time, there has been a consistent increase in research activity, reflecting a growing academic and societal interest in this field.

**Table 1.** Publication trends over time.

Year	Number of publications	*P*
1912	1	0.08
1928	1	0.08
1935	1	0.08
1946	2	0.15
1965	2	0.15
1966	2	0.15
1967	3	0.23
1968	1	0.08
1971	2	0.15
1972	6	0.46
1973	10	0.76
1974	16	1.22
1975	18	1.38
1976	14	1.07
1977	9	0.69
1978	6	0.46
1979	4	0.31
1980	3	0.23
1981	4	0.31
1982	5	0.38
1984	1	0.08
1985	5	0.38
1986	6	0.46
1987	3	0.23
1988	7	0.53
1989	5	0.38
1990	8	0.61
1991	4	0.31
1992	5	0.38
1993	4	0.31
1994	5	0.38
1995	5	0.38
1996	5	0.38
1997	10	0.76
1998	12	0.92
1999	11	0.84
2000	12	0.92
2001	17	1.30
2002	20	1.53
2003	28	2.14
2004	14	1.07
2005	24	1.83
2006	41	3.13
2007	17	1.30
2008	41	3.13
2009	41	3.13
2010	46	3.51
2011	58	4.43
2012	42	3.21
2013	59	4.51
2014	64	4.89
2015	54	4.13
2016	47	3.59
2017	51	3.90
2018	51	3.90
2019	59	4.51
2020	66	5.04
2021	67	5.12
2022	51	3.90
2023	58	4.43
2024	69	5.27
2025	6	0.46

**Figure 1.**
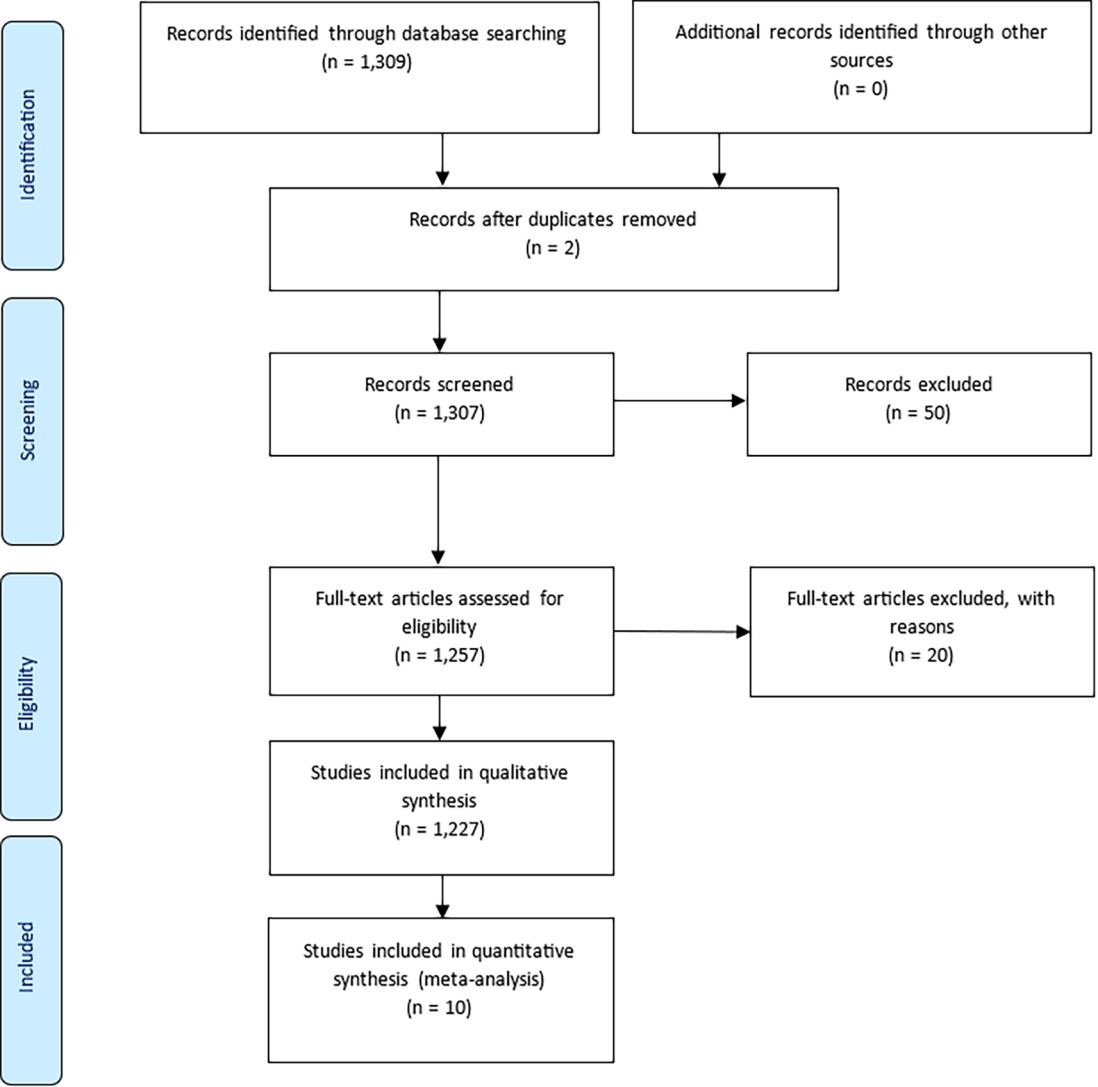
PRISMA flow diagram showing the selection process of articles included in the bibliometric review, adapted from Moher et al., (2010).

*3.1.1 1912–1960s: Sparse and inconsistent output*

Research activity during this period was minimal. Only a few isolated publications emerged in 1912, 1928, and 1935. From 1946 to the late 1960s, the number of annual publications remained between one and three, suggesting limited academic focus or resource availability during these decades.

*3.1.2 1970s–1990s: Gradual growth*

The early 1970s saw a modest rise in scholarly output, from 2 publications in 1971 to 18 by 1975. This increase may reflect advances in research methodologies, data accessibility, or funding opportunities ( Ninkov et al., 2021). During the 1980s and early 1990s, publication counts stabilized between 4 and 10 per year, indicating a gradual normalization of research in this area despite occasional fluctuations.

*3.1.3 2000–2010: Marked acceleration*

The early 2000s marked a significant upsurge in research production. Between 2000 and 2005, annual publications rose from 12 to 24, and by 2006, the figure jumped to 41. This acceleration continued through 2008 and 2009, with 41 publications each year, followed by 46 in 2010. The surge is likely linked to technological advancements, digital access to academic databases, and increased institutional support for research ( Kalyani, 2024).

*3.1.4 2011–2020: Peak research activity*

The field reached a period of high productivity during this decade. In 2011, 58 publications were recorded, and this number remained consistently high in the years that followed. The highest output was in 2021, with 67 publications, closely followed by 66 in 2020. This trend indicates robust global academic engagement, driven by collaborative efforts, increased funding, and growing policy relevance ( Kwiek, 2021).

*3.1.5 2021–2025: Sustained out with slight variability*

Following 2021, publication numbers remained strong, with over 50 documents annually. The year 2024 reached a new peak with 69 publications. The lower number in 2025 (six publications) is likely due to the partial nature of the data for that year. Overall, the increasing trend in publication volume—especially since the early 2000s—signals sustained interest and growing scholarly momentum.

This trend also suggests that earlier decades were constrained by factors such as limited research funding, technological barriers, and restricted international collaboration ( Arbour et al., 2021). The acceleration in the 21st century reflects the influence of policy shifts, infrastructure expansion, and multidisciplinary interest in the psychological rehabilitation of inmates.

Future studies may benefit from investigating the underlying causes of these temporal shifts in research output and how these patterns correspond to changes in global prison reform agendas, funding mechanisms, and collaborative networks.

### 3.2 Geographic distribution and collaboration network of authors

*3.2.1 Geographic distribution of authors*

Table 2 displays the distribution of authors based on institutional affiliation. The United States leads with 11 affiliated authors, followed by Germany and the United Kingdom. This indicates a Western dominance in prisoner resilience and motivation research. Leading contributors include institutions such as the University of New South Wales, California State University, and the University of Antwerp, emphasizing the role of well-resourced academic centers in advancing this domain ( Abdel-Salam & Kilmer, 2023).

**Table 2.** Geographic distribution of authors.

Affiliation	Count	*P*
United States	11	28.95
Germany	3	7.89
School of Engineering and Information Technology, University of New South Wales, Australian Defence Force Academy, Canberra, ACT, Australia	2	5.26
Safer Custody Group, HM Prison Service, United Kingdom	2	5.26
California State University, Fresno, United States	2	5.26
Bath Spa University, United Kingdom	2	5.26
Tambov State Technical University, Tambov, Russian Federation	2	5.26
United Kingdom	2	5.26
Department of Philosophy, Marquette University, Milwaukee, WI 53210-1881, PO Box 1881, United States	1	2.63
School of Psychology, University of Newcastle, NSW, Australia; National Drug Research Institute, Curtin University of Technology, WA, Australia; New South Wales Justice Health, NSW, Australia	1	2.63
Service de médecine interne, Maladies infectiousness, UCSA, Centre hospitalier régional Félix-Guyon, 97400 Saint-Denis, Reunion; UCSA de Lille-Loos-Sequedin, Centre hospitalier Régional de Lille, 59000 Lille, France; UCSA de Loos-Lez-Lille de Lille-Sequedin, UHSI, Centre hospitalier régional universitaire de Lille, 59000 Lille, France; Service de médecine polyvalente, Centre hospitalier régional Félix-Guyon, 97400 Saint-Denis, Reunion	1	2.63
Section of Trauma Studies, Division of Psychological Medicine and Psychiatry, Institute of Psychiatry, United Kingdom; Istanbul Center for Behavior Research and Therapy (ICBRT/DABATEM), Istanbul, Turkey	1	2.63
Florida State University, Tallahassee, FL, United States; Florida Department of Corrections, Tallahassee, FL, United States	1	2.63
Department of Psychology, Cardiff School of Health Sciences, University of Wales Institute Cardiff (UWIC), Llandaff Campus, CF52YB, Western Avenue, Cardiff, United Kingdom	1	2.63
Department of Psychology, University of Colorado at Boulder, United States; Center for AIDS Intervention Research (CAIR), Medical College of Wisconsin, United States; Department of Psychology, University of Nevada at Reno, United States; Department of Psychology, Center on Alcoholism, Substance Abuse, and Addictions (CASAA), University of New Mexico, United States	1	2.63
Treatment Research Institute, 600 Public Ledger Bldg., 150 South Independence Mall West, Philadelphia, PA 19106; Treatment Research Institute, University of Pennsylvania	1	2.63
Florida State University, College of Social Work, Tallahassee, FL 32306, 296 Champions Way, United States; University of Kansas, Lawrence, United States; University of Denver, Colorado, United States	1	2.63
Department of Psychiatry, University of New Mexico School of Medicine, Albuquerque, NM, United States; Correctional Medical Services, NM, United States; Santa Fe, NM 87505, 2442 Cerrillos Road #105, United States	1	2.63
University of Alabama at Birmingham, United States; Medical University of South Carolina, United States; Johns Hopkins University, United States	1	2.63
University of Antwerp, Faculty of Applied Economics, B-2000 Antwerpen, Prinsstraat 13, Belgium	1	2.63

Institutions like the Section of Trauma Studies at the Institute of Psychiatry (UK) and the Istanbul Center for Behavior Research and Therapy (Turkey) reflect increasing global interest and collaboration. U.S.-based institutions such as Florida State University and the University of New Mexico highlight the integration of criminal justice and mental health research in correctional contexts.

Although contributions from developed nations dominate, emerging entries from countries like Russia and Belgium suggest a growing international scope. However, the underrepresentation of African, Latin American, and some Asian institutions suggests a need for broader geographic inclusion to better reflect diverse prison systems and cultural contexts.

**Figure 2.**
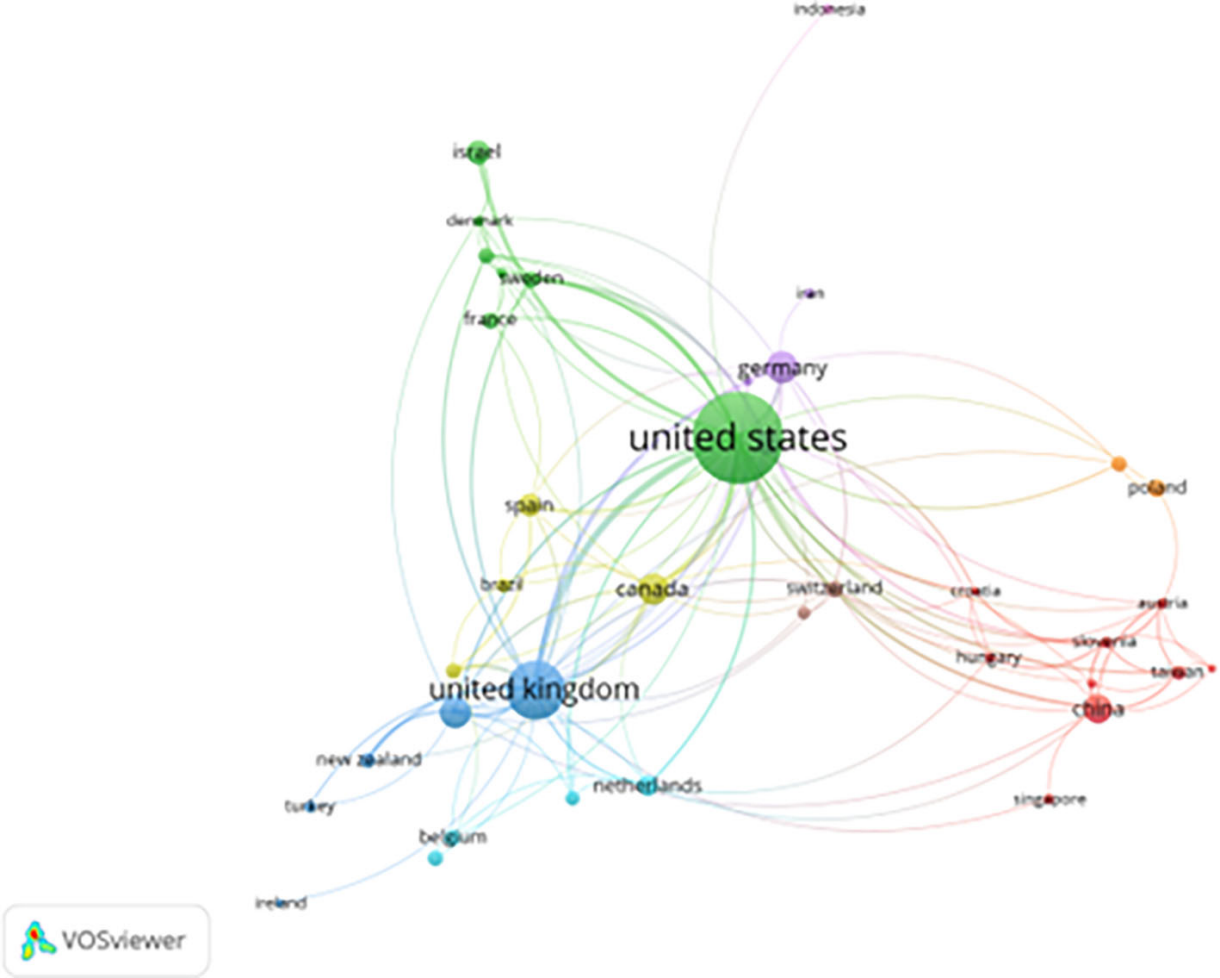
Country collaboration network in prisoner resilience and motivation research (VOSviewer visualization).

Figure 2 illustrates the international collaboration network in prisoner resilience and motivation research. The map reveals that the United States, United Kingdom, Germany, Canada, and China represent the most active contributors and hosts of interconnected research networks. The U.S. dominates both North American and global collaboration, supported by strong federal funding and well-established university-based research centers focused on criminology, psychology, and corrections ( Batastini et al., 2024).

European collaboration clusters—particularly among the UK, Germany, France, and the Netherlands—reflect shared correctional policies and EU-supported research initiatives in justice and rehabilitation. Similarly, clusters in East Asia involving China, Singapore, and Taiwan suggest growing academic interest in applying psychological resilience frameworks within correctional settings facing challenges such as overcrowding and recidivism.

Emerging participation from countries like Indonesia, Iran, and Turkey is evident, though these nations remain more loosely integrated into global networks. Barriers such as language, limited research funding, and distinct correctional philosophies may hinder deeper collaboration.

The thickness and density of connecting lines on the map represent the frequency and strength of international partnerships. Strong links between North America and Europe indicate frequent joint projects and shared research agendas. In contrast, countries from Africa, South America, and the Middle East are largely absent or marginal in the network, highlighting regional underrepresentation in global discourse on prison rehabilitation.

This collaboration map underscores the urgent need to enhance research integration across underrepresented regions. Greater global inclusion would promote culturally grounded insights into resilience and motivation, fostering a more holistic understanding of prisoner rehabilitation across diverse penal systems.

*3.2.2 Collaboration networks among authors*

Table 3 shows the most collaborative authors based on co-authorship frequency. Stein L.A.R., Sarchiapone M., and Ward T. each appeared in seven collaborative works, indicating their central role in research networks. Authors such as Roy A., Boone C., and Brochu S. also demonstrate high collaboration levels, contributing to the interdisciplinary integration of psychology, criminology, and behavioral sciences.

**Table 3.** Collaboration networks among authors.

Author	Collaboration count	*P*
Stein L.A.R.	7	7.29
Sarchiapone M.	7	7.29
Ward T.	7	7.29
Roy A.	6	6.25
Boone C.	5	5.21
Moore J.L.	5	5.21
Brochu S.	5	5.21
Perc M.	5	5.21
Knight K.	5	5.21
Stuewig J.	4	4.17
Kiehl K.A.	4	4.17
Martin R.A.	4	4.17
Winder B.	4	4.17
Hoyt R.E.	4	4.17
Chen X.	4	4.17
Pinto da Costa M.	4	4.17
Carli V.	4	4.17
Day A.	4	4.17
Sánchez A.	4	4.17
Stams G.J.J.M.	4	4.17

These collaboration patterns suggest strong research clusters, with co-authors forming long-term academic partnerships across various subfields—including correctional mental health, rehabilitation strategies, and motivational interventions ( Mejia et al., 2021). However, the network also reveals fragmented segments, where early-career researchers or those from underrepresented regions may be working in isolation.

**Figure 3.**
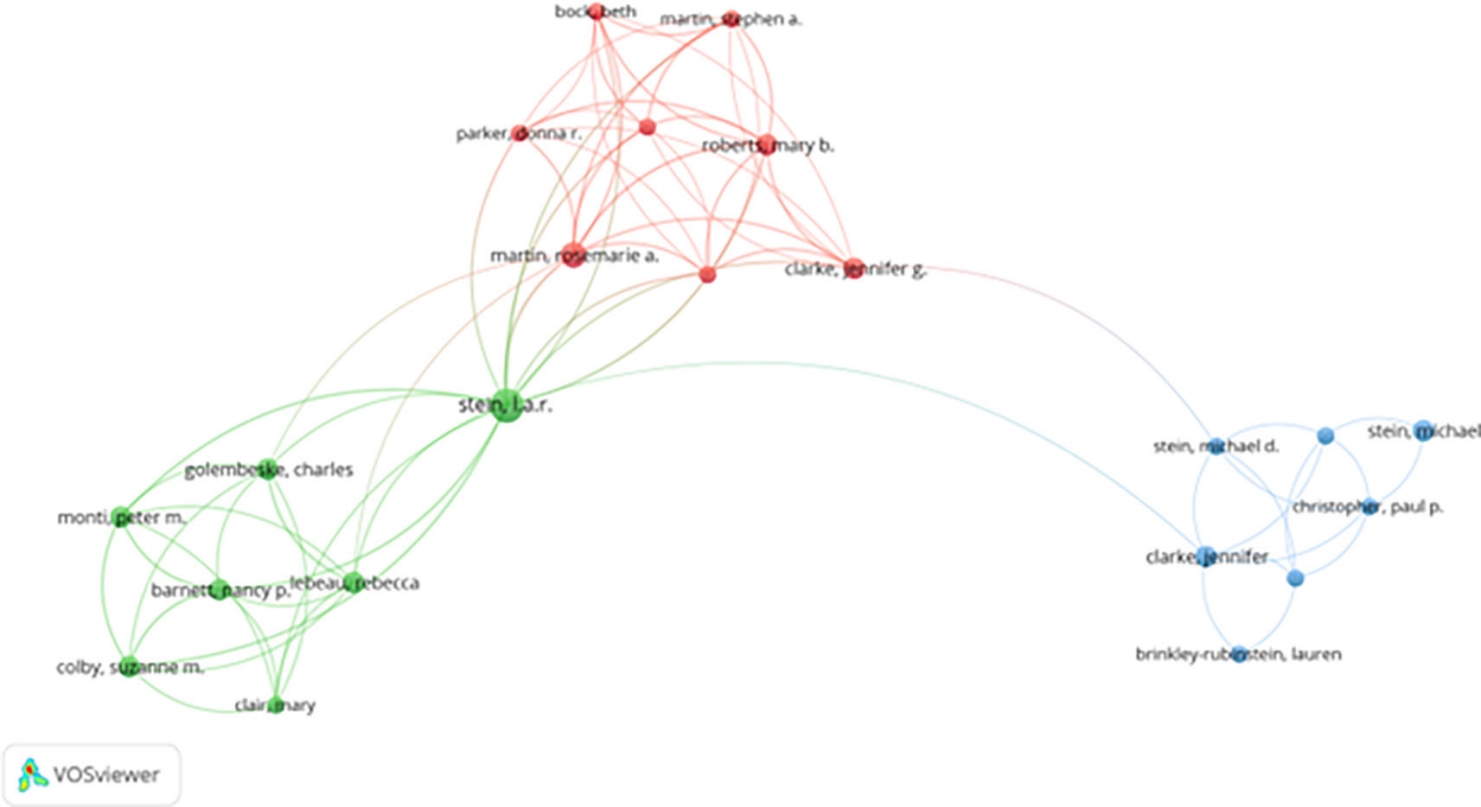
VosViewer analysis on author co-authorship network in prisoner resilience and motivation research.

Figure 3 (co-authorship network via VOSviewer) illustrates these clusters, with several prominent collaborative hubs centered around authors like Stein and Martin. These clusters show thematic specialization, such as drug addiction recovery, psychological therapy, and motivational enhancement in correctional settings.

Despite strong internal collaboration, limited cross-cluster integration suggests missed opportunities for broader theoretical exchange. Strengthening interdisciplinary and cross-regional collaboration could foster innovation and methodological diversity in prisoner rehabilitation research.

*3.2.3 Institutional contributions and collaborations*

**Figure 4.**
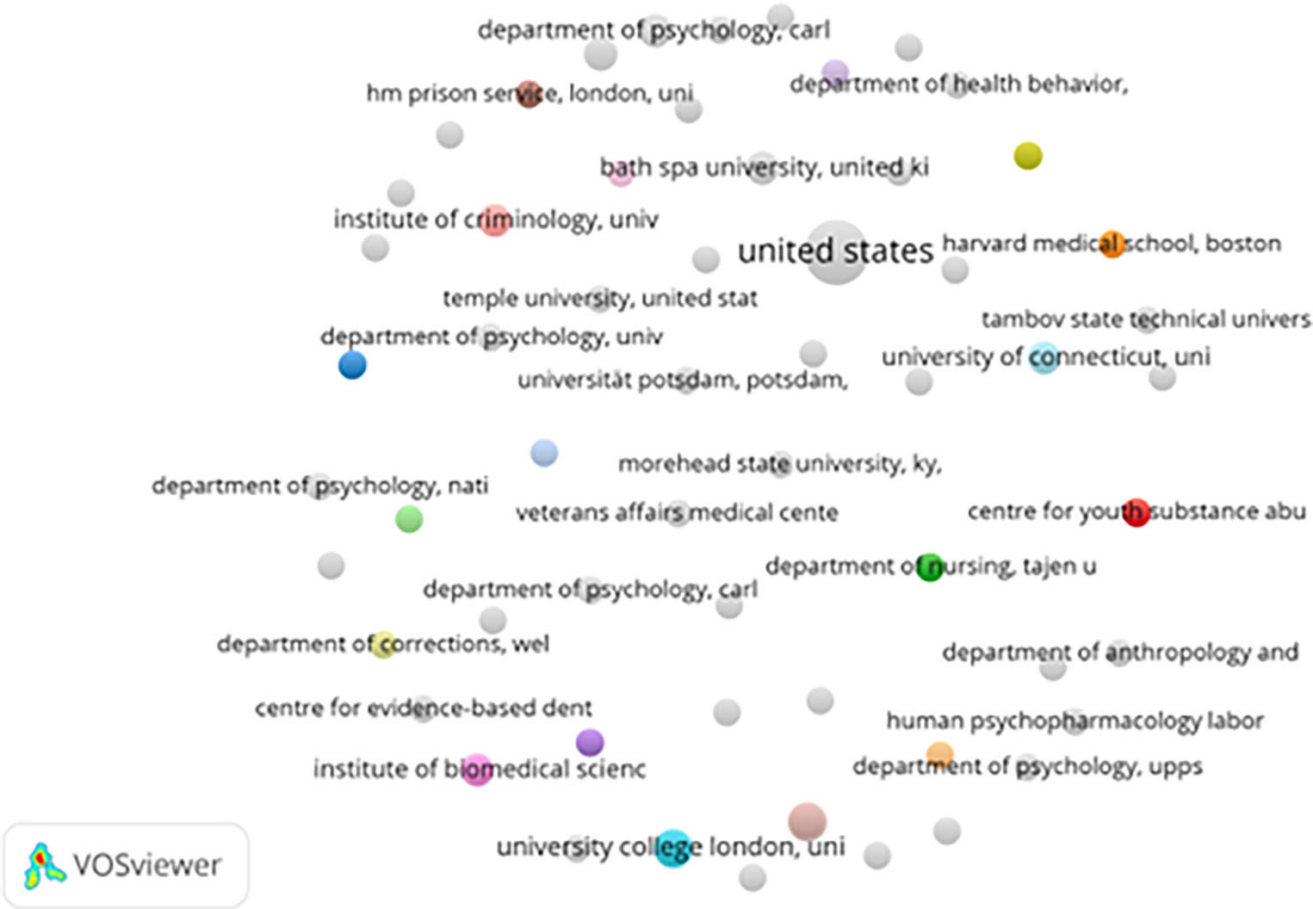
VosViewer analysis on institutional collaboration network in prisoner resilience and motivation research.

Figure 4 presents the institutional collaboration network, highlighting contributions from leading academic centers. Institutions like Temple University, Harvard Medical School, and University College London demonstrate strong interconnectivity. These institutions often collaborate on projects spanning mental health, criminology, and rehabilitative psychology, reflecting the field’s interdisciplinary nature.

Institutional linkages tend to cluster geographically. North American and European institutions dominate the collaboration landscape, while institutions from developing countries remain largely disconnected. This imbalance suggests that prison rehabilitation research is still heavily influenced by Western perspectives ( Hedges et al., 2021).

Government-linked institutions such as the Veterans Affairs Medical Center (USA) and HM Prison Service (UK) signal practical applications of research findings. These collaborations bridge academic work with policy and practice, supporting evidence-based correctional strategies.

Nevertheless, the absence of institutional partners from Africa, Latin America, and parts of Asia limits the global applicability of current findings. Expanding research partnerships through joint funding, academic exchanges, and collaborative studies could address this gap and promote more inclusive correctional rehabilitation frameworks.

In summary, Section 3.2 demonstrates that while prisoner resilience and motivation research is growing, it remains concentrated in high-income countries and established research hubs. Promoting international, cross-disciplinary collaboration is essential for advancing global understanding and developing culturally responsive interventions.

### 3.3 Thematic areas and research focus

*3.3.1 All keywords*

**Figure 5.**
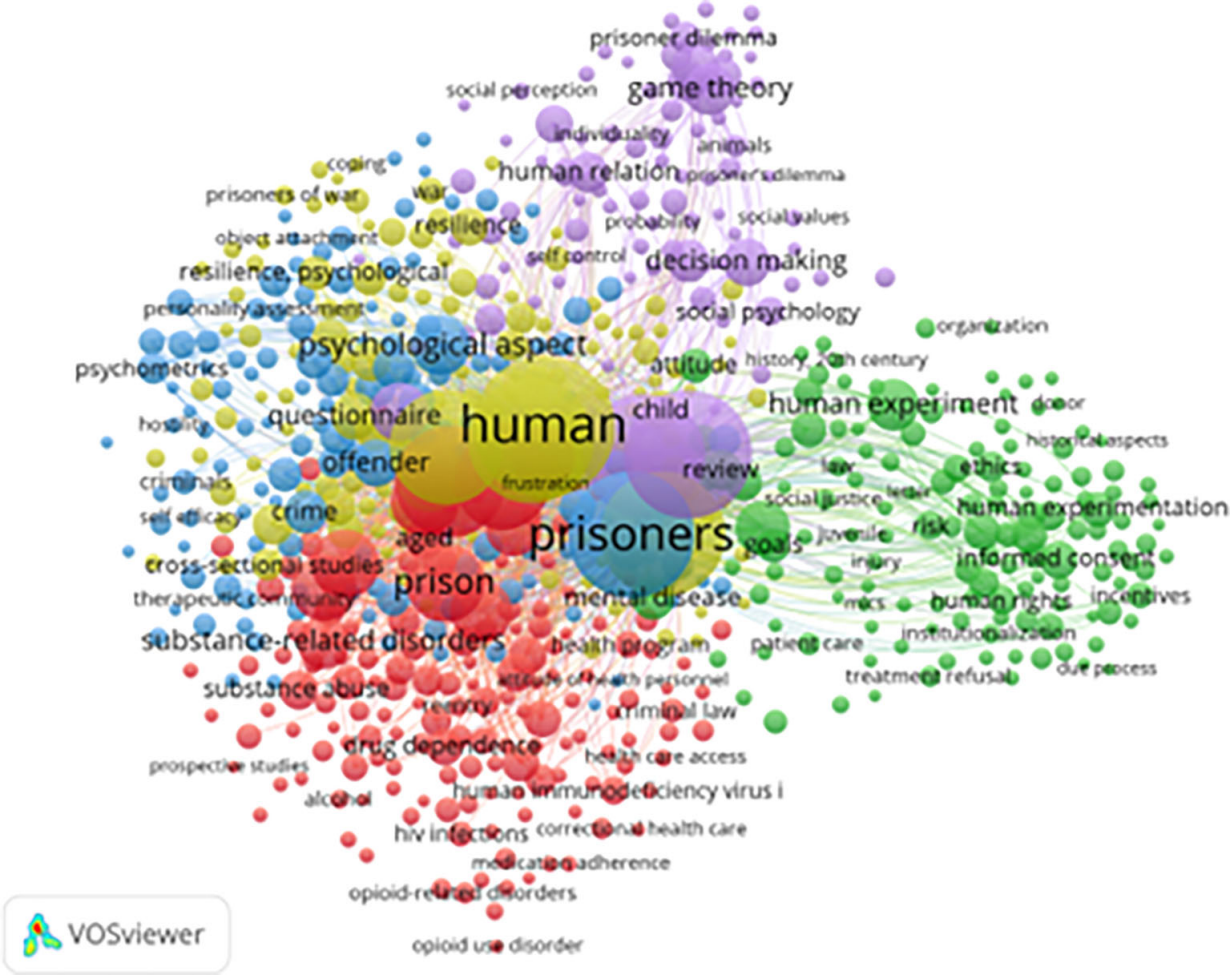
VosViewer analysis on keyword co-occurrence network in resilience and motivation research. Note: Minimum number of occurrences of keyword 7 of the 6221 keywords, 650 meet the thresholds.

Figure 5 presents a keyword co-occurrence network generated using VOSviewer, highlighting dominant themes and research intersections in the study of resilience and motivation among prisoners. A total of 650 keywords met the minimum threshold for inclusion (out of 6,221 total keywords with at least seven occurrences).

The most frequently linked terms include “prisoners,” “resilience,” “motivation,” “coping,” “psychological aspects,” “self-efficacy,” “drug addiction,” and “rehabilitation.” The strength of connections between these keywords suggests an interdisciplinary approach, bridging correctional psychology, criminology, addiction recovery, and behavioral health ( Radhakrishnan et al., 2017; Bukar et al., 2023).

Thematic clusters in the network reveal four major research domains:

1) **Psychosocial Resilience (Blue Cluster):** Focuses on emotional regulation, self-efficacy, decision-making, and coping strategies. These studies emphasize how incarcerated individuals psychologically adapt to the prison environment.
2) **Substance Abuse and Rehabilitation (Red Cluster):** Includes terms like “addiction,” “opioid use disorder,” and “dependence,” reflecting a strong research emphasis on addressing substance-related issues within prison populations ( Schulz et al., 2023).
3) **Legal and Ethical Issues (Green Cluster):** Keywords such as “criminal law,” “human rights,” “informed consent,” and “institutionalization” suggest attention to correctional ethics, justice reform, and prisoner rights.
4) **Behavioral Decision-Making (Yellow Cluster):** Featuring terms like “game theory,” “prisoner’s dilemma,” and “decision-making,” this cluster highlights research applying behavioral economics and psychology to prison-based social interactions.

Other important terms such as “HIV prevention,” “treatment adherence,” and “healthcare access” indicate growing awareness of health inequities within correctional settings. The appearance of keywords such as “women prisoners” and “gender” marks the emergence of more inclusive research on vulnerable populations.

Despite these strengths, gaps remain. Keywords related to vocational training, post-incarceration employment, and economic reintegration are notably underrepresented. In addition, there is a lack of emphasis on cultural adaptation and ethnic diversity, suggesting the need for more context-sensitive studies, particularly from non-Western environments.

This analysis underscores a transition in focus from punitive models to rehabilitative and rights-based frameworks, while also highlighting areas that warrant further investigation—especially gender-specific interventions and post-release support systems.

*3.3.2 Keyword co-occurrence and emerging trends*

**Figure 6.**
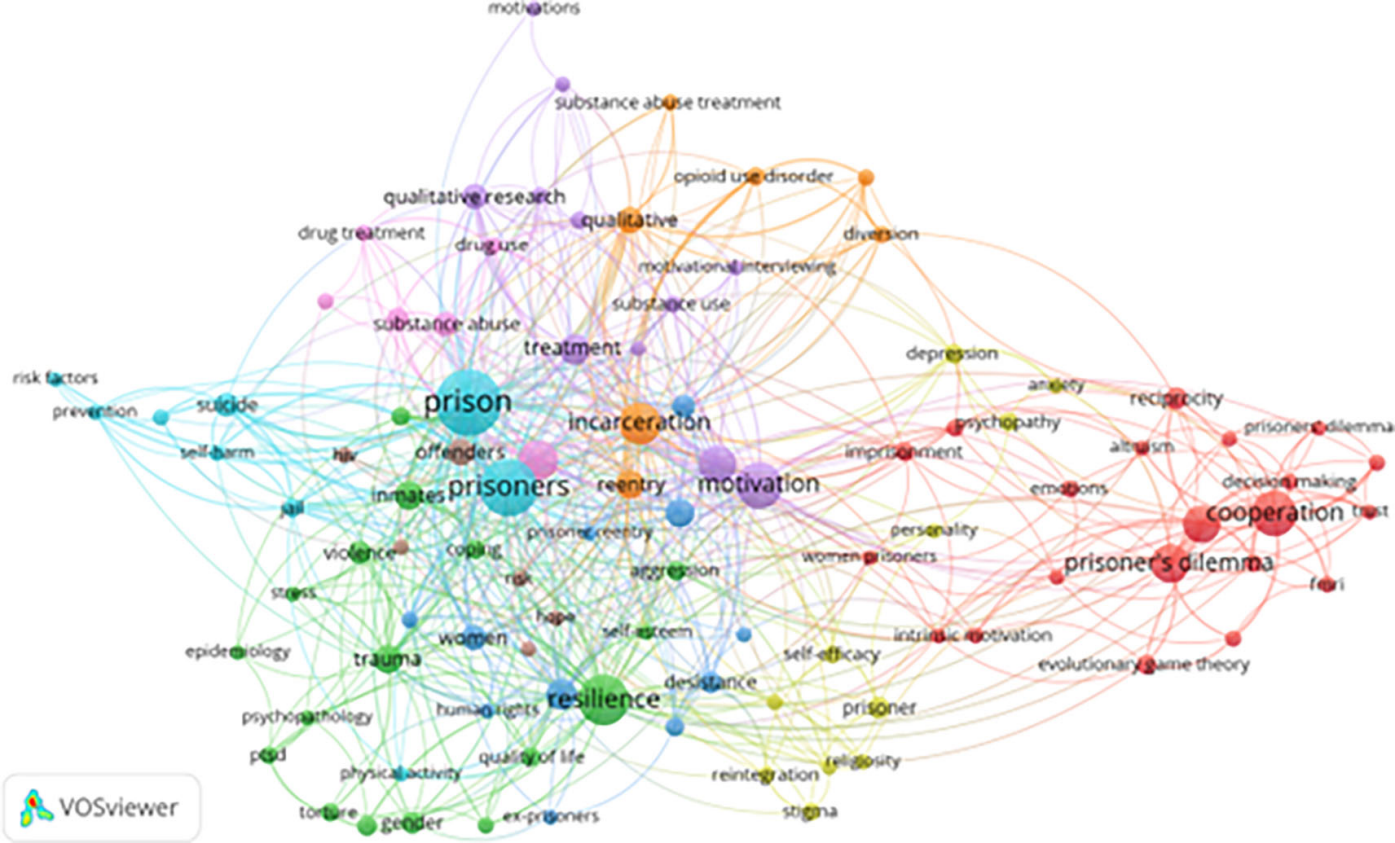
VosViewer analysis on keyword co-occurrence and emerging trends. Note: Minimum number of occurrences of a keyword 5 of the 2678 keywords, 99 meet the thresholds.

Figure 6 displays a temporal co-occurrence network based on 99 keywords (from a total of 2,678 keywords with at least five mentions). This visualization highlights not only established research themes but also emerging trends.

The most prominent emerging clusters include:

1) **Psychological Resilience & Coping Strategies:** Keywords such as “trauma,” “self-esteem,” “quality of life,” and “human rights” dominate this area. Recent literature increasingly focuses on subgroup-specific resilience strategies, particularly for women and minority inmates.
2) **Substance Abuse Recovery:** Terms like “opioid use disorder,” “pharmacotherapy,” and “motivational interviewing” illustrate the continued emphasis on addiction as both a cause and consequence of incarceration. This area links closely with motivational theories and CBT-based recovery models, particularly those implemented through therapeutic community approaches that emphasize structured peer support and motivational change among incarcerated individuals ( Sacks et al., 2012).
3) **Post-Incarceration Reintegration:** Keywords like “stigma,” “hope,” “goal setting,” and “faith-based rehabilitation” reflect efforts to address the challenges prisoners face after release. There is growing interest in community support systems and individual agency during reentry.
4) **Decision-Making Models:** “Behavioral economics,” “trust,” “risk-taking,” and “prisoner’s dilemma” are increasingly used to explain inmate choices and social behavior within the institutional setting. These terms reflect a nuanced understanding of how incarcerated individuals assess consequences and navigate prison dynamics.
5) **Ethical and Legal Concerns:** Frequent appearance of terms like “medical ethics,” “criminal justice reform,” and “prison health disparities” indicates a growing critique of systemic injustices within correctional systems.

This keyword network highlights a critical shift in correctional research—from institutional control and punishment to rehabilitation, resilience, and restorative justice. However, several crucial aspects—such as cultural adaptation, technological innovation in interventions, and long-term reentry outcomes—remain underexplored.

*3.3.3 Index keywords*

**Figure 7.**
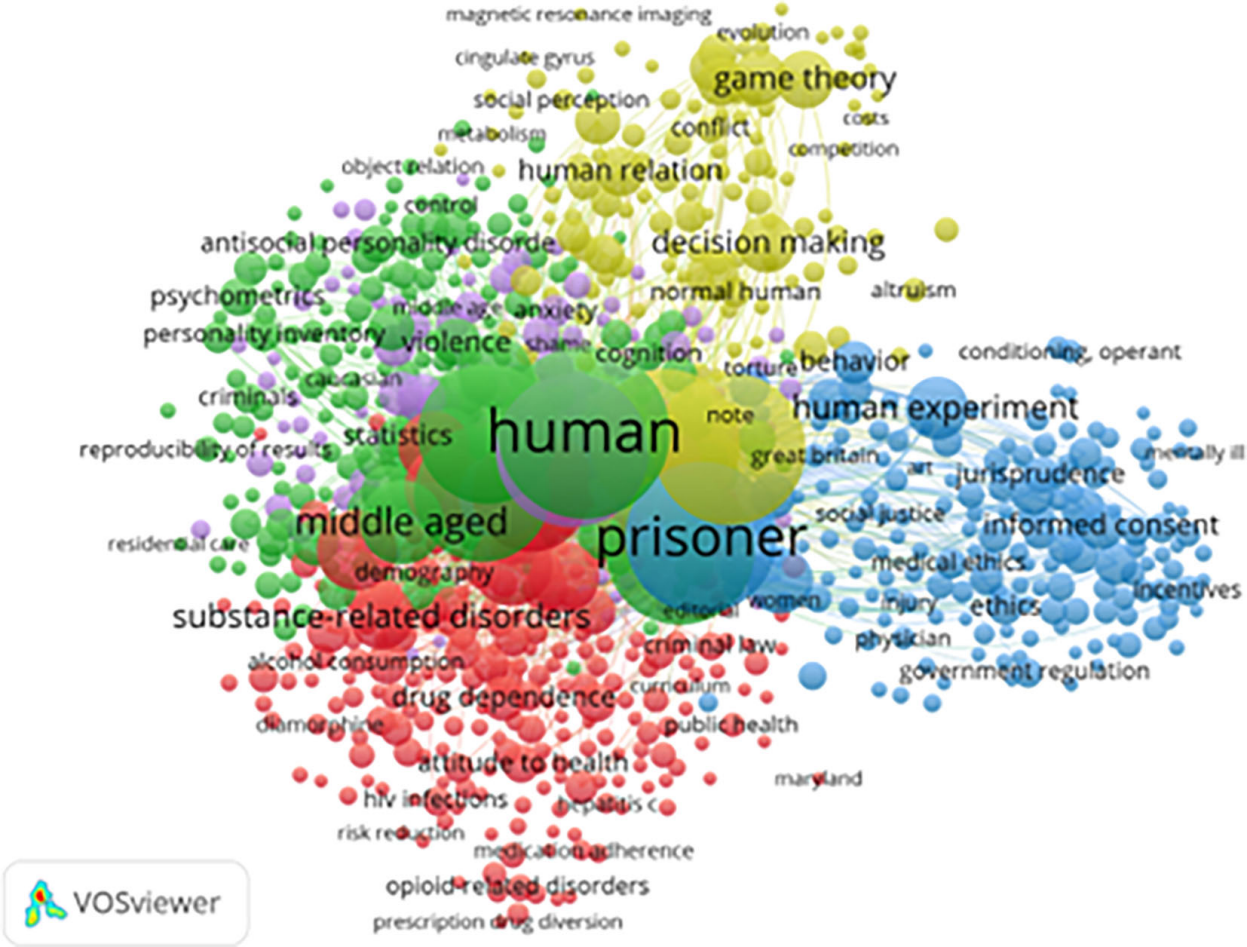
VosViewer analysis on index keywords in resilience and motivation research among prisoners. Note: Minimum number of occurrences of a keyword 5 of the 4203 keywords, 824 meet the thresholds.

Figure 7 presents index keyword clusters that further classify core topics in the field. A total of 824 keywords (from 4,203 with at least five mentions) met the threshold for inclusion. These clusters echo and refine earlier findings:

1) **Green Cluster (Psychological Themes):** Includes terms like “anxiety,” “personality,” and “psychometrics.” This cluster suggests sustained interest in how individual traits relate to resilience and behavioral adaptation in prison.
2) **Red Cluster (Health and Addiction):** Dominated by “drug use,” “alcohol,” “medication adherence,” and “HIV,” this cluster reflects ongoing concern for the health outcomes and therapeutic needs of incarcerated populations.
3) **Blue Cluster (Legal and Ethical Dimensions):** Featuring “jurisprudence,” “informed consent,” and “government regulation,” this area underscores the legal and policy-level discourse on ethical incarceration and prisoners’ rights.
4) **Yellow Cluster (Decision-Making and Institutional Behavior):** Involving terms like “game theory,” “cooperation,” “cognitive processing,” and “trust,” this cluster points to research on inmate social dynamics and institutional interactions.

Despite comprehensive coverage, significant gaps remain:

1) Gender-Specific Interventions are underrepresented, with limited focus on women, LGBTQ+, or elderly inmates.
2) Cross-Cultural Resilience Frameworks are rare, reflecting the dominance of Western-centric perspectives in existing literature.
3) Post-Release Economic Reintegration and employment readiness are infrequent themes, though they are critical to long-term desistance from crime ( Chandrasekaran et al., 2024).

In conclusion, keyword analysis across all levels shows that while research has diversified and matured, more culturally inclusive, gender-sensitive, and post-release-focused studies are needed. Advancing this field requires sustained interdisciplinary collaboration among psychologists, criminologists, public health experts, and policymakers to create context-specific, effective interventions for incarcerated individuals worldwide.

*3.3.4 Temporal and thematic evolution of research*

The evolving color transition also indicates an increasing research convergence between resilience, motivation, and rehabilitation domains—signalling a more holistic correctional paradigm emphasizing personal growth, social connection, and reintegration. In addition, co-authorship mapping shows that collaborative networks have become more globally interconnected since 2015, particularly involving scholars from the United Kingdom, the United States, Australia, Malaysia, and South Africa. This globalization of research networks mirrors the broadening theoretical lens through which resilience and motivation among incarcerated individuals are now understood.

**Figure 8.**
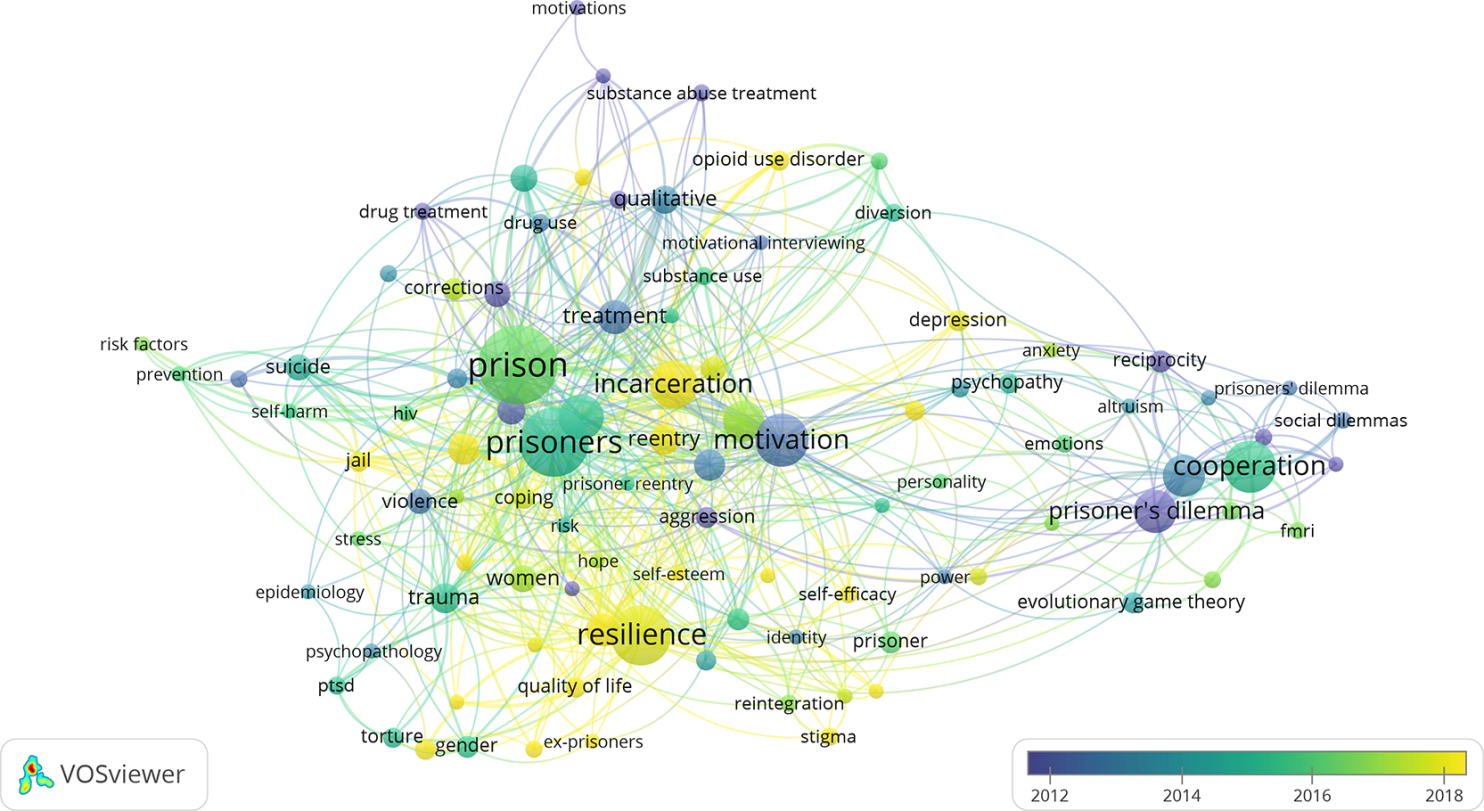
Temporal evolution of research themes (1912–2024).

Overlay visualization map generated using VOSviewer (version 1.6.19) based on keyword co-occurrence analysis. The color gradient represents the average publication year, showing the chronological progression of key research themes from early psychological adaptation studies (blue) to more recent emphases on motivation, resilience, and reintegration (yellow). Larger nodes indicate higher keyword frequency, while line thickness represents the strength of co-occurrence links. The map highlights the temporal convergence between resilience, motivation, and rehabilitation research, reflecting the field’s increasing interdisciplinarity and theoretical diversification.

### 3.4 Citation analysis and research influence

**Figure 9.**
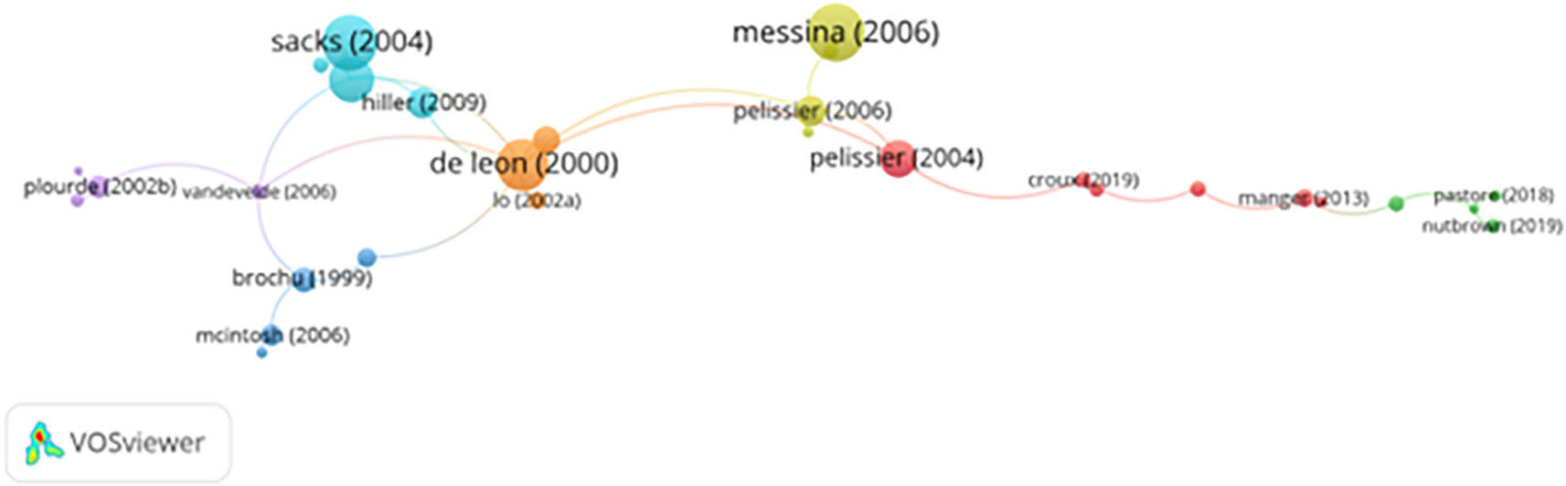
VosViewer analysis on citation analysis and research influence. Note: Minimum number of citations of a document: 3 of the 1309 documents, 907 meet the thresholds.

Figure 9 illustrates the citation network of key publications within prisoner resilience and motivation research. Central nodes represent highly cited works, and the size and thickness of the connecting lines reflect citation volume and strength of scholarly influence.

Seminal works by De Leon (2000), Peters et al. (2004), Messina et al. (2006), Manger et al. (2010) and Borseth et al. (2025) stand out as foundational to the field. Subsequent studies by Pelissier et al. (2003) and Messina et al. (2006) further illustrated how educational and therapeutic interventions enhance prisoners’ intrinsic motivation and contribute to reduced recidivism through evidence-based rehabilitation frameworks. These contributions collectively shifted correctional approaches from punitive deterrence to rehabilitative and motivational paradigms supported by cognitive-behavioral principles ( Christensen & Hesstvedt, 2024). Recent citation activity has pivoted toward reintegration research. Authors like King & Smith (2024) and Sahoo et al., (2025) explore how post-release experiences—such as employment access, social support, and mental health—impact long-term resilience and recidivism. These studies highlight the growing importance of holistic reentry frameworks in contemporary correctional research. Scholars such as Chouhy et al. (2020) and Link et al. (2019) have examined how employment access, social support, and mental health services influence long-term desistance from crime. These studies signal a maturation of the field toward holistic reentry frameworks, reflecting an interdisciplinary engagement with sociology, criminology, and public health.

*3.4.1 Gaps and structural asymmetries*

Despite these advancements, the citation network reveals persistent structural and epistemic imbalances that shape the global production of knowledge in this field:

1. Underrepresentation of non-Western scholarship:
The vast majority of highly cited works originate from North American and European institutions. This concentration reflects not only differences in research capacity and funding but also historical dominance in criminological theorizing. Consequently, much of the field’s conceptual vocabulary—such as *rehabilitation*, *desistance*, and *reentry*—is rooted in Western correctional paradigms, potentially overlooking culturally distinct understandings of transformation and moral reform.
2. Lack of comparative cross-cultural analyses:
Few cited studies explore how resilience and motivation vary across different legal systems, cultural frameworks, or economic contexts. Integrating perspectives from Asia, Africa, Latin America, and the Middle East would deepen the field’s explanatory power and enhance its relevance for regions where incarceration is influenced by poverty, religion, or community-based justice systems.
3. Epistemic hierarchies in citation practices:
Citation visibility is strongly tied to journal indexing and linguistic accessibility. English-language dominance within global publishing ecosystems perpetuates citation bias, marginalizing research disseminated through regional or non-indexed journals. As Bosworth (2023) notes, this dynamic sustains a form of epistemic coloniality, where Global North frameworks are reproduced as universal standards of criminological knowledge.

**Journal co-citation patterns and interdisciplinary convergence**

**Figure 10.**
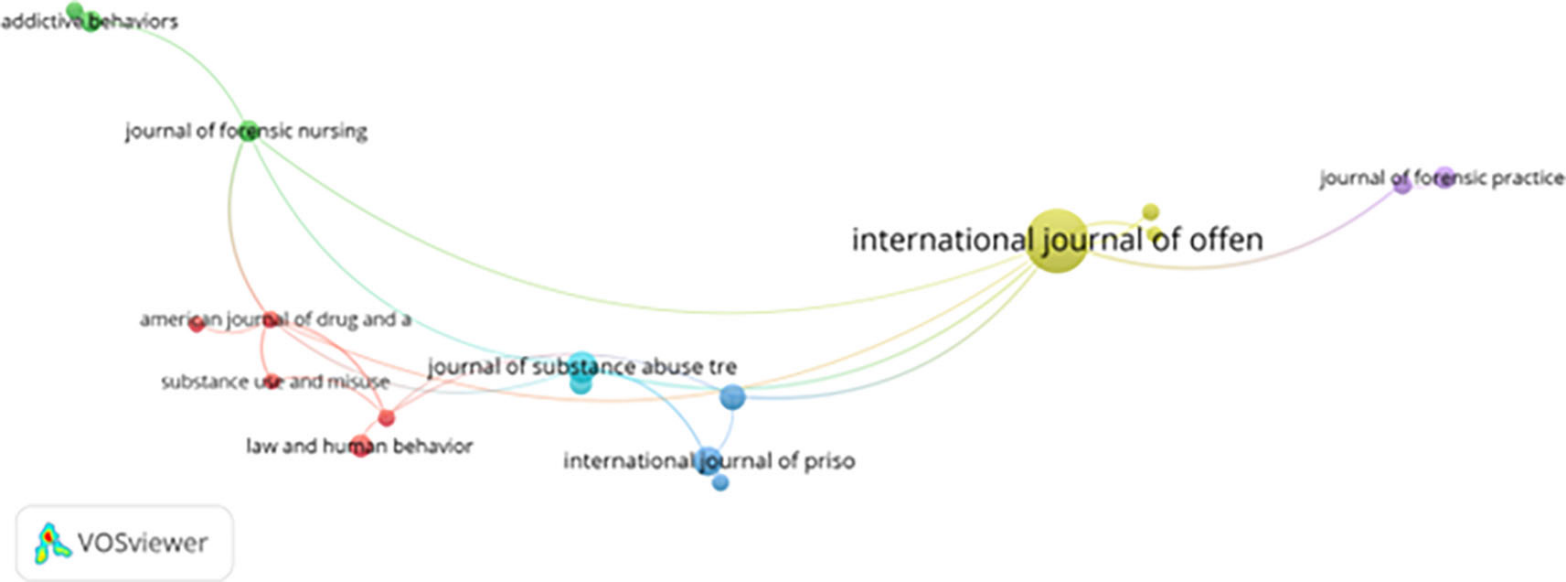
VosViewer analysis on journal co-citation analysis of the most prominent academic sources. Note: Minimum number of documents of a source 5 of the 784 sources, 32 meet the thresholds.

Figure 10 further demonstrates journal co-citation patterns, identifying key academic sources that shape the discipline. Journals such as the Journal of Offender Therapy and Comparative Criminology, Addictive Behaviors, American Journal of Drug and Alcohol Abuse, and Law and Human Behavior serve as primary platforms for publishing and citing foundational research.

These journals collectively reflect an interdisciplinary focus encompassing:

1. Addiction recovery and harm reduction,
2. Forensic psychology and behavioral assessment, and
3. Rehabilitation strategies and criminal justice reform.

The frequent co-citation of literature from psychology, criminology, and public health confirms the transdisciplinary nature of prisoner motivation and resilience studies. However, the limited visibility of regionally focused journals—particularly those from the Global South—raises concerns about citation bias and scholarly imbalance, potentially constraining innovation in localized correctional practices.

*3.4.2 Interpretive and sociopolitical reflection*

The observed concentration of citations within Western academic systems reflects not only material advantages—such as access to funding and international databases—but also ideological continuities tied to the postcolonial history of penal systems. Criminological theories developed in the Global North have often been exported to other contexts without adequate cultural adaptation, reinforcing one-dimensional understandings of rehabilitation that prioritize institutional control over community-based transformation and cultural embeddedness ( Carrington et al., 2018).

Drawing from Braithwaite’s (2002) notion of responsive regulation and Garland’s (2018) analysis of penal modernity, it becomes evident that the global diffusion of these ideas also entails moral and cultural translation. In many non-Western contexts, concepts of resilience and motivation are intertwined with religious belief systems, collective responsibility, and familial obligations—dimensions rarely captured in mainstream Western literature. Recognizing these pluralities is essential for developing a decolonized, context-sensitive criminology that values indigenous, faith-based, and community-driven understandings of prisoner transformation.

*3.4.3 Synthesis and practical relevance*

In summary, citation and co-citation analyses reveal the evolution of the field from early research on therapeutic communities and addiction recovery to contemporary emphases on reintegration, mental health, and justice reform. While the field benefits from a strong empirical and theoretical foundation, its future advancement depends on structural inclusivity, cultural sensitivity, and knowledge democratization.

Encouraging South–South research collaborations, expanding access to open bibliometric repositories, and diversifying editorial representation in leading journals would help redress current imbalances. In turn, such measures could promote a more equitable global exchange of ideas and foster rehabilitation frameworks that reflect the lived realities of prisoners worldwide.

## 4. Conclusions

This bibliometric review provides the first comprehensive mapping of more than a century of scholarship (1912–2024) on resilience and motivation among incarcerated populations. By synthesizing publication trends, thematic clusters, citation networks, and institutional collaboration patterns, the study delineates how this field has evolved from individual-centered psychological models to broader, socially embedded understandings of transformation and reintegration. The findings reveal that research on prisoner resilience and motivation has matured into a vibrant interdisciplinary domain spanning psychology, criminology, social work, education, and public health.

However, the analysis also uncovers structural and epistemic asymmetries that shape the production and circulation of criminological knowledge. Western institutions continue to dominate both publication output and citation influence, reflecting historical legacies of colonial knowledge systems and global disparities in academic visibility. Such imbalances risk reproducing universalist models of rehabilitation that may not align with the cultural, spiritual, and socioeconomic realities of incarcerated populations in the Global South. Addressing these inequities requires an intentional movement toward decolonizing correctional research, embracing pluralistic epistemologies, and foregrounding locally grounded experiences of recovery, moral growth, and resilience.

The study’s insights hold several practical and policy implications. Policymakers and practitioners can use bibliometric evidence to identify dominant frameworks, assess research gaps, and design more contextually appropriate interventions. For example, integrating motivation- and resilience-based models into correctional education, peer-support, and post-release programs could enhance rehabilitation outcomes and reduce recidivism. At the same time, strengthening international collaboration networks—particularly South–South partnerships—can democratize access to research resources, increase methodological diversity, and foster innovation in evidence-based correctional practices.

From a theoretical perspective, the evolution of resilience and motivation research mirrors the transition from pathology-oriented rehabilitation to strength-based and transformative justice approaches. This shift underscores the importance of situating individual change within structural and relational contexts, emphasizing the roles of community, family, and social inclusion in sustaining desistance from crime. Future research should continue to integrate critical criminology, postcolonial theory, and responsive justice frameworks to better understand how power, inequality, and institutional structures shape opportunities for prisoner transformation.

In conclusion, the field of prisoner resilience and motivation stands at a critical juncture. The next stage of its development must move beyond disciplinary and geographical boundaries, embracing methodological transparency, cultural reflexivity, and ethical inclusivity. By expanding its theoretical reach and engaging underrepresented regions, this scholarship can contribute not only to improved correctional outcomes but also to a more humane, equitable, and context-sensitive vision of justice and rehabilitation worldwide.

## Ethical considerations

Not applicable.

## Data Availability

The dataset generated and analysed during the current study is openly available at: https://doi.org/10.6084/m9.figshare.29321222.v1 [ Hussin (2025)]. This project contains following datafiles:
1. scopus (2).csv 2. PRISMA checklist Data are available under the terms of the Creative Commons Zero “No rights reserved” data waiver (CC0 1.0 Public domain dedication).
