# A bibliometric review of research on resilience, motivation and prisoners, 1912-2024

**DOI:** 10.12688/f1000research.164019.3

**Published:** 2026-01-06

**Authors:** Zalmizy Hussin, Md Zawawi Abu Bakar, Mohd Ahsani A.Malek, NoorSuzana Mohd Shariff, Siti Rohana Ahmad

**Affiliations:** 1 School of Applied Psychology, Social Work and Policy, Universiti Utara Malaysia, Sintok, Kedah, Malaysia; 2 Department of Community Health, Advanced Medical and Dental Institute, Universiti Sains Malaysia, Kepala Batas, Pulau Pinang, Malaysia; 3 Family Health Department, Kedah State Health Department, Alor Setar, Malaysia

**Keywords:** Resilience, motivation, prisoners, rehabilitation, bibliometric analysis

## Abstract

**Background:**

Research on resilience and motivation among incarcerated populations has expanded substantially over the past century, reflecting a broader shift in penal philosophy from punitive control toward rehabilitation and reintegration. However, this literature remains fragmented across disciplines, conceptually heterogeneous, and geographically uneven, with a strong concentration in Western contexts. This limits theoretical integration and constrains the global applicability of existing models.

**Methods:**

A bibliometric analysis was conducted on 1,309 publications indexed between 1912 and 2024. Co-occurrence, citation, collaboration, and temporal overlay analyses were performed using VOSviewer to map thematic structures, intellectual influences, and patterns of knowledge production. Inclusion criteria targeted peer-reviewed and scholarly works addressing resilience and/or motivation in correctional settings.

**Results and Discussion:**

The analysis reveals a temporal shift from early psychological adjustment and behavioral control models toward integrated frameworks emphasizing resilience, motivation, rehabilitation, and post-release reintegration. Emerging themes include trauma-informed care, addiction recovery, human rights, and reentry processes, while gender-specific, cross-cultural, and socioeconomic reintegration perspectives remain underdeveloped. Citation and collaboration networks demonstrate strong institutional concentration in North America and Western Europe, reflecting structural and epistemic asymmetries that shape dominant theoretical paradigms. Although interdisciplinary integration has increased, comparative and culturally grounded research remain limited.

**Conclusions:**

This review synthesizes a century of research into a coherent intellectual map, identifying key trends, gaps, and structural imbalances in the field. The findings highlight the need for more inclusive, culturally responsive, and structurally informed research frameworks that integrate psychological, institutional, and social dimensions of rehabilitation. Advancing this agenda is essential for developing correctional policies and interventions that are both empirically robust and globally relevant.

## 1. Introduction

### 1.1 Research background

Research on resilience and motivation occupies a central position across psychology, criminology, sociology, and public health because both constructs are foundational to understanding how individuals adapt to adversity and sustain behavioral change under conditions of constraint ( Adams, 2022; Schulz et al., 2023). Resilience refers to a dynamic process of psychological adaptation through which individuals maintain or regain mental health in the face of stress, trauma, or structural disadvantage ( Tomaszewski, 2023; Uddin et al., 2012). Motivation, in contrast, concerns the processes that initiate, direct, and sustain goal-oriented behavior, including both intrinsic drivers (e.g., meaning, autonomy) and extrinsic incentives ( Deci & Ryan, 2012; Schulz et al., 2023).

While often studied separately, resilience and motivation are theoretically and empirically interdependent. Resilience provides the psychological capacity to withstand and recover from adversity, whereas motivation supplies the behavioral energy necessary to engage in adaptive or change-oriented action. In high-stress institutional environments, resilience can be understood as a *precondition* for sustained motivation, while motivation functions as a *mechanism* through which resilience is translated into constructive behavior. This reciprocal relationship becomes particularly salient in contexts characterized by chronic stress, restricted autonomy, and limited personal agency — conditions that typify incarceration ( Passas, 2024).

Prisons represent an extreme institutional setting in which both resilience and motivation are continuously tested. Incarcerated individuals are exposed to cumulative stressors including social isolation, stigma, loss of autonomy, institutional violence, and uncertainty about the future ( Curley et al., 2025). These stressors frequently interact with pre-existing vulnerabilities such as mental illness, trauma histories, and substance dependence, intensifying psychological burden and complicating rehabilitation efforts ( Batistich et al., 2025; Klinoff et al., 2018). Under such conditions, resilience is essential for psychological survival and emotional regulation, while motivation is required for participation in rehabilitative activities such as education, therapy, and vocational training ( Paquette et al., 2025).

Importantly, resilience and motivation do not operate independently within prisons; rather, they form a mutually reinforcing system. Individuals with higher resilience are more capable of sustaining engagement in programs that require long-term effort and delayed rewards, while motivational engagement itself can strengthen resilience by enhancing self-efficacy, perceived control, and future orientation. In correctional settings, this interdependence shapes key outcomes such as program completion, behavioral compliance, psychological well-being, and post-release adjustment.

Over the past century, correctional philosophy has gradually shifted from predominantly punitive models toward frameworks emphasizing rehabilitation, reintegration, and psychological support ( Ahmed et al., 2025; Durkin, 2025). This transformation has been accompanied by a growing scholarly focus on psychosocial mechanisms — including resilience and motivation — that mediate how individuals respond to incarceration and how institutional environments influence personal change ( Dewey et al., 2024; Powrie, 2025). Within this literature, resilience is increasingly framed as a protective factor against institutional harm, while motivation is conceptualized as a driver of rehabilitative engagement and desistance from crime.

Despite this expanding body of work, research remains fragmented across disciplines, theoretical traditions, and empirical domains. Studies differ in how they conceptualize resilience and motivation, how they operationalize these constructs, and how they locate them within broader institutional and cultural contexts. Moreover, much of the existing literature is concentrated in Western settings, raising questions about the generalizability of dominant models to correctional systems shaped by different legal traditions, cultural norms, and resource constraints.

A systematic, bibliometric mapping of this field is therefore necessary to clarify how resilience and motivation have been conceptualized, connected, and studied over time within prison research, to identify dominant themes and theoretical orientations, and to reveal structural gaps and regional asymmetries in knowledge production. By situating resilience and motivation within the specific institutional ecology of prisons — rather than treating them as abstract psychological traits — this review aims to advance a more integrated and context-sensitive understanding of how psychological adaptation and behavioral change unfold under conditions of confinement.

### 1.2 Review of literature

Early scholarship on resilience emerged within developmental psychology and focused on how individuals adapt to adversity associated with poverty, neglect, or abuse ( David & Towl, 2021). Seminal longitudinal studies emphasized the role of emotional regulation, problem-solving capacity, and access to stable social support in facilitating adaptive outcomes ( Whitehead et al., 2014). This work established resilience not as a fixed trait but as a dynamic, context-dependent process shaped by individual, relational, and structural factors ( Pamungkas et al., 2023). Contemporary resilience research extends beyond childhood adversity to encompass institutional, cultural, and socio-economic environments, recognizing that adaptive capacity is embedded within broader social systems.

Within correctional contexts, resilience has been conceptualized as the psychological capacity to manage the chronic stressors associated with confinement, including loss of autonomy, exposure to coercive authority, social isolation, and institutional violence. Empirical studies indicate that structured psychological interventions—such as cognitive behavioral therapy (CBT), mindfulness-based programs, and peer-support initiatives—can enhance emotional regulation, reduce distress, and promote adaptive coping among incarcerated populations ( Carmo et al., 2024; Derlic, 2022; Harding-White et al., 2024; Riley et al., 2019). These interventions do not merely alleviate symptoms but also create conditions that enable individuals to sustain engagement with rehabilitative processes over time.

Motivation research has developed in parallel, with increasing attention to how incarcerated individuals initiate and sustain participation in programs that require effort, persistence, and delayed gratification. While early motivational frameworks emphasized need fulfillment and self-efficacy, more recent work grounded in Self-Determination Theory (SDT) highlights the centrality of autonomy, competence, and relatedness in fostering durable behavioral engagement ( Moller et al., 2014; Ryan & Reeve, 2021; Ntsame Sima et al., 2024). In correctional settings, where autonomy is structurally constrained, motivation becomes particularly fragile and dependent on institutional practices, staff-prisoner relationships, and program design.

Research suggests that motivation is not only an outcome of rehabilitative engagement but also a mediator between institutional conditions and behavioral change. Motivational interviewing, goal-setting interventions, and strengths-based programming have been associated with improved participation in education, vocational training, and treatment programs ( Pandya, 2022). These findings indicate that motivation functions as a critical mechanism through which institutional environments influence individual trajectories. This is consistent with findings by Pastwa-Wojciechowska and Guzińska (2024), who reported that higher self-efficacy among male prisoners is significantly associated with greater motivation and sustained participation in addiction treatment programs.

An emerging body of work explicitly links resilience and motivation as mutually reinforcing processes. Resilience supports the emotional and cognitive stability required to persist in long-term programs, while motivation provides the directional force that translates adaptive capacity into sustained action. Studies show that higher resilience is associated with greater self-efficacy, persistence, and goal commitment, while motivational engagement strengthens resilience by reinforcing agency, future orientation, and perceived control ( Huang et al., 2020; Navickienė & Vasiliauskas, 2024; Schulz et al., 2023). In prison contexts, this reciprocal relationship is central to rehabilitation because individuals must both withstand institutional stress and actively invest in change-oriented behaviors.

Despite this conceptual and empirical progress, several limitations characterize existing literature. First, the majority of studies are concentrated in Western, high-income countries, particularly North America and Western Europe. This geographic concentration restricts the generalizability of dominant models and obscures how resilience and motivation operate within correctional systems shaped by different legal traditions, cultural norms, and resource constraints. Comparative criminological research indicates that institutional goals, rehabilitation philosophies, and definitions of personal change vary substantially across regions, suggesting that Western frameworks may not adequately capture global diversity in correctional practice.

Second, much of the literature remains focused on individual-level psychological processes, with comparatively limited attention to institutional, structural, and policy-level determinants. Factors such as prison overcrowding, staff culture, access to programs, sentence length, and post-release support infrastructure shape both resilience and motivation yet are rarely integrated into analytic models. This limit understanding of how institutional design and governance structures condition individual psychological outcomes.

Third, although post-release reintegration is frequently identified as a key policy objective, relatively little empirical work examines how resilience and motivation developed during incarceration translate into employment, mental health, social integration, and desistance after release. Without this longitudinal perspective, it remains unclear which forms of psychological adaptation have lasting rehabilitative value and which are merely adaptive within the prison environment itself.

Taking together, these limitations indicate that the field is theoretically fragmented, geographically uneven, and analytically incomplete. While numerous studies address components of resilience and motivation, there has been no comprehensive synthesis of how these constructs have been conceptualized, connected, and empirically examined across time, disciplines, and regions. A systematic bibliometric review is therefore warranted to map the intellectual structure of the field, identify dominant theoretical and methodological trends, reveal neglected areas and populations, and clarify how scholarly attention has evolved in response to shifting correctional paradigms.

By situating resilience and motivation within the institutional ecology of prisons and tracing their treatment across more than a century of scholarship, this review seeks to provide a structured foundation for future theory development, empirical research, and policy design in correctional rehabilitation.

### 1.3 Study objectives

This bibliometric review aims to analyze trends, methodologies, and thematic developments in research on resilience and motivation among incarcerated individuals between 1912 and 2024. The specific objectives are to:

1. Track the historical development of scholarly work on resilience and motivation in correctional contexts, including major milestones and shifts in theoretical and empirical focus.
2. Identify and evaluate leading countries, institutions, and scholars contributing to this field, as well as patterns of collaboration and influence.
3. Analyze dominant research themes, keyword structures, and citation networks to guide future research and highlight neglected areas.

By integrating fragmented and multidisciplinary literature, this review maps the intellectual evolution of the field and informs the development of evidence-based interventions and correctional policies aimed at improving mental health, reducing recidivism, and supporting successful reintegration into society.

## 2. Materials and Methods

### 2.1 Source identification

This study utilized the Scopus database as the primary source of bibliographic data due to its comprehensive coverage in the social sciences, psychology, and public health disciplines. Compared to alternatives like Web of Science, Scopus offers broader indexing and improved accessibility to high-quality scholarly publications ( Pranckutė, 2021; Tennant, 2020). The review included a diverse range of documents, including journal articles, books, book chapters, and conference proceedings. To ensure inclusivity, the search strategy used the keywords: “resilience” OR “motivation” AND “prisoners.” No start year was specified in the search query, allowing for the inclusion of the earliest available records.

The study adhered to the PRISMA (Preferred Reporting Items for Systematic Reviews and Meta-Analyses) guidelines to ensure transparency in the identification, screening, and selection of documents ( Moher et al., 2010; Pham & Le, 2024). An initial Scopus search yielded 1,309 documents. After removing two duplicates, 1,307 records remained for screening. A title and abstract review excluded 50 documents unrelated to prisoner resilience or motivation. A further 20 records were excluded during full-text assessment for lacking methodological relevance. This process resulted in the inclusion of 1,227 articles for qualitative synthesis, and 10 for quantitative synthesis (meta-analysis).

### 2.2 Data analysis

The final dataset comprised 1,309 Scopus-indexed publications focusing on *prisoner resilience* and *motivation* from 1912 to 2024. Extracted bibliographic elements included authorship, publication year, source title, institutional affiliation, country, keywords, and citation metrics. Both descriptive and bibliometric techniques were applied to identify trends, collaboration patterns, and structural relationships within the field.

Descriptive statistics were first employed to examine the temporal distribution of publications, highlighting distinct periods of scholarly growth and thematic diversification. Institutional and country-level affiliations were analyzed to identify leading contributors and regions of research concentration. Citation and co-citation analyses were then used to determine the intellectual structure of the field, pinpointing influential scholars and foundational works ( Ding et al., 2014; Robledo-Giraldo et al., 2023).

Bibliometric mapping and visualization were conducted using VOSviewer software (version 1.6.19) developed by Van Eck and Waltman. The full dataset was exported from Scopus in *CSV format* with complete bibliographic information. To ensure transparency and reproducibility, a multi-stage analytical protocol was implemented as follows:

1. Data Cleaning: Duplicate records, incomplete metadata, non-English titles, errata, and editorial materials were removed manually using Microsoft Excel to ensure data consistency and quality control.
2. Keyword Standardization: Synonymous or variant keywords were consolidated to avoid redundancy (e.g., *resilience* and *resiliency*; *motivation* and *motivational factors*; *inmates* and *prisoners*). The *thesaurus* function in VOSviewer was utilized to merge similar terms and unify terminology across the dataset.
3. Software Configuration: Analyses were performed using standardized parameters to enhance comparability:
  ○ *Minimum number of keyword occurrences:* 5
  ○ *Resolution:* 0.85
  ○ *Normalization method:* Association Strength
  ○ *Visualization types:* Network, Overlay, and Density maps
4. Thematic and Temporal Mapping: Co-occurrence and co-authorship analyses were performed separately for keywords, authors, and institutions. The overlay visualization function was used to trace the temporal evolution of major research themes, applying a year-based gradient color scheme to visualize how topics have shifted from early studies on coping and adjustment to contemporary emphases on rehabilitation, transformation, and reintegration.
5. Reliability and Reproducibility: All data-handling steps, search queries, and software settings were archived as a supplementary file (see *Data Availability Statement*) to enable replication and extension by future researchers.

The integration of these analytical approaches provided a comprehensive evaluation of the field’s development, capturing both structural and temporal dimensions of scholarly productivity. This combination of descriptive and network-based techniques enabled the identification of dominant thematic clusters, emerging interdisciplinary linkages, and geographic disparities in research output—thereby fulfilling the study’s objectives of mapping intellectual influence, collaboration, and thematic evolution ( Lim et al., 2024).

This methodological triangulation not only ensures analytical robustness but also situates bibliometric patterns within broader theoretical interpretations of resilience and motivation in correctional contexts.

### 2.3 Inclusion and exclusion criteria

Rigorous inclusion and exclusion criteria were applied to ensure the relevance, consistency, and methodological integrity of the bibliometric dataset. Eligible publications included peer-reviewed empirical studies, theoretical articles, systematic or narrative reviews, book chapters, and conference proceedings that substantively addressed resilience and/or motivation in relation to incarcerated populations.

Only English-language documents were included. This restriction was applied to ensure linguistic consistency in bibliographic metadata, enable accurate keyword standardization and citation analysis, and facilitate transparent replication by an international research audience. The temporal scope covered publications from 1912 to 2024 in order to capture both the historical emergence and contemporary development of the field. Initial relevance was assessed through title and abstract screening, followed by full-text assessment when necessary.

Publications were excluded if they lacked substantive empirical or conceptual engagement with resilience or motivation in correctional contexts. This included editorial notes, opinion pieces, brief reports without analytic content, letters to the editor, errata, and retracted publications. Records with incomplete bibliographic information—such as missing author names, publication titles, or source identifiers—were also excluded to preserve the accuracy and reliability of network and citation analyses.

These criteria were designed to balance inclusivity with analytical rigor, ensuring that the final dataset reflected a coherent and methodologically robust body of literature suitable for mapping intellectual trends, thematic structures, and collaboration patterns within resilience and motivation research in correctional settings.

### 2.4 Reliability

The study ensured reliability through standardized procedures and adherence to established bibliometric protocols ( Babu & Kohli, 2023). The selection of Scopus as the data source enhanced data validity, given its peer-reviewed and indexed content.

The use of clearly defined inclusion and exclusion criteria minimized selection bias and enhanced reproducibility. Compliance with PRISMA guidelines ensured transparency in the review process, from initial identification to final synthesis.

VOSviewer software provided a consistent and replicable platform for network and trend analysis, including co-authorship, co-citation, and keyword mapping. Duplicate and incomplete records were eliminated, and only verified entries were retained in the final dataset.

These methodological safeguards contributed to the robustness, consistency, and credibility of the study’s findings, offering a dependable foundation for future research in this field.

## 3. Results and Discussion

### 3.1 Year of publication

Table 1 presents the temporal distribution of publications on resilience and motivation among prisoners between 1912 and 2025. The overall pattern reveals a long period of sparse activity followed by a pronounced and sustained expansion beginning in the early 2000s, indicating a gradual transformation of this topic from a marginal concern into a distinct and increasingly institutionalized field of inquiry.

**Table 1.** Publication trends over time.

Year	Number of publications	*P*
1912	1	0.08
1928	1	0.08
1935	1	0.08
1946	2	0.15
1965	2	0.15
1966	2	0.15
1967	3	0.23
1968	1	0.08
1971	2	0.15
1972	6	0.46
1973	10	0.76
1974	16	1.22
1975	18	1.38
1976	14	1.07
1977	9	0.69
1978	6	0.46
1979	4	0.31
1980	3	0.23
1981	4	0.31
1982	5	0.38
1984	1	0.08
1985	5	0.38
1986	6	0.46
1987	3	0.23
1988	7	0.53
1989	5	0.38
1990	8	0.61
1991	4	0.31
1992	5	0.38
1993	4	0.31
1994	5	0.38
1995	5	0.38
1996	5	0.38
1997	10	0.76
1998	12	0.92
1999	11	0.84
2000	12	0.92
2001	17	1.30
2002	20	1.53
2003	28	2.14
2004	14	1.07
2005	24	1.83
2006	41	3.13
2007	17	1.30
2008	41	3.13
2009	41	3.13
2010	46	3.51
2011	58	4.43
2012	42	3.21
2013	59	4.51
2014	64	4.89
2015	54	4.13
2016	47	3.59
2017	51	3.90
2018	51	3.90
2019	59	4.51
2020	66	5.04
2021	67	5.12
2022	51	3.90
2023	58	4.43
2024	69	5.27
2025	6	0.46

**Figure 1.**
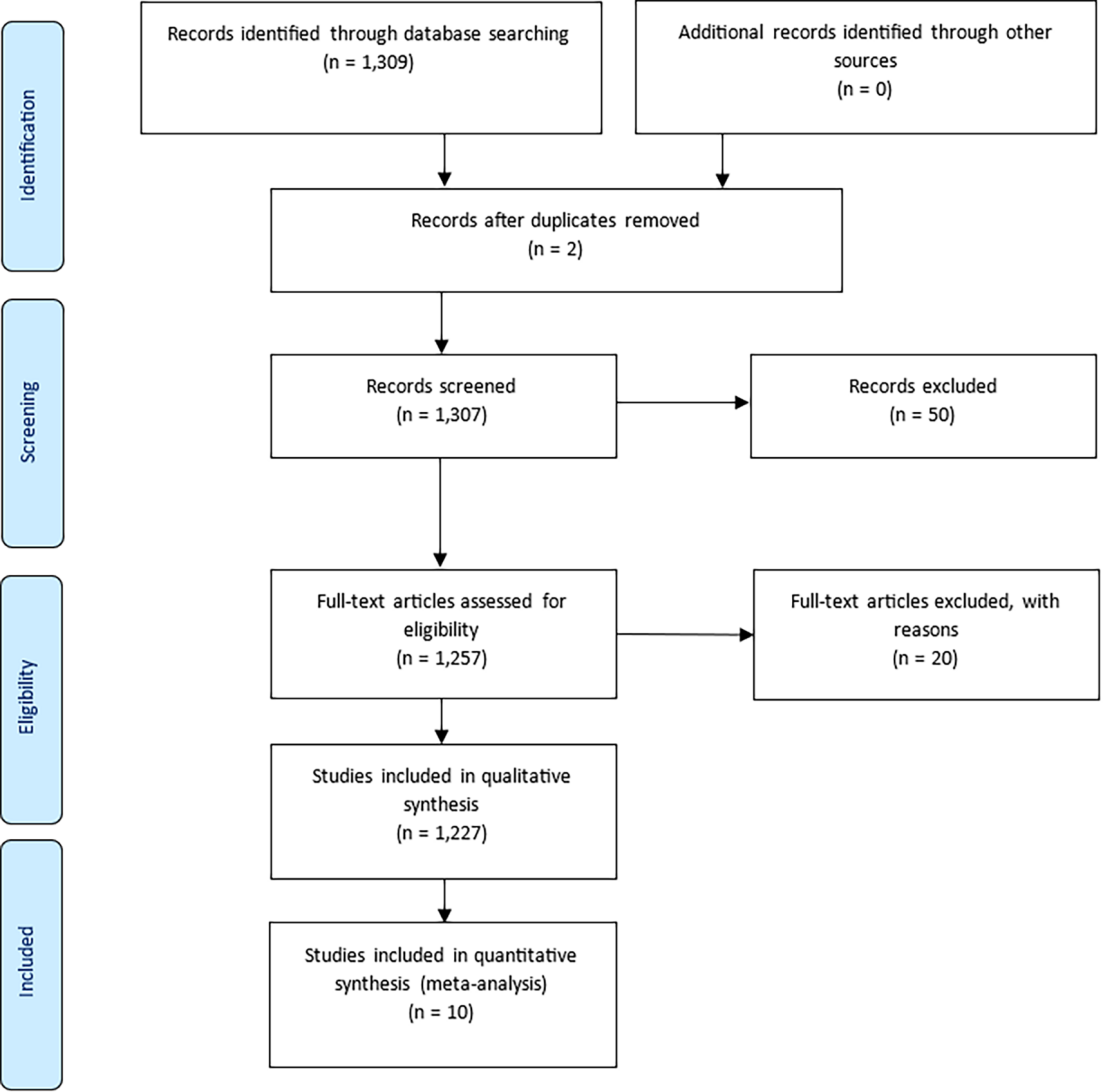
PRISMA flow diagram showing the selection process of articles included in the bibliometric review, adapted from Moher et al. (2010).

*3.1.1 1912–1960s: Sparse and inconsistent output*

During this early period, scholarly attention to resilience and motivation in correctional contexts was minimal. Only isolated publications appeared in 1912, 1928, and 1935, and annual output between 1946 and the late 1960s rarely exceeded three publications. This limited production likely reflects the dominance of punitive and deterrence-oriented penal paradigms during much of the twentieth century, as well as restricted methodological tools, limited access to data, and low institutional prioritization of rehabilitation research.

*3.1.2 1970s–1990s: Gradual growth*

The early 1970s marked the beginning of a modest but sustained increase in research output, rising from two publications in 1971 to eighteen in 1975. This growth coincides with broader shifts in criminology and social policy toward rehabilitation, offender treatment, and psychosocial explanations of criminal behavior. Advances in research methods, data availability, and funding mechanisms likely facilitated this expansion ( Ninkov et al., 2021). Throughout the 1980s and 1990s, publication levels stabilized at moderate levels, suggesting the consolidation of the field rather than rapid innovation.

*3.1.3 2000–2010: Marked acceleration*

The early 2000s represent a clear inflection point. Between 2000 and 2006, annual output more than tripled, rising from 12 to 41 publications, and remained elevated thereafter. This acceleration aligns with the expansion of digital academic infrastructures, bibliographic databases, and interdisciplinary research funding, as well as growing political and institutional attention to rehabilitation, mental health, and recidivism reduction ( Kalyani, 2024).

*3.1.4 2011–2020: Peak research activity*

The period from 2011 onward reflects sustained high productivity, with annual publications consistently exceeding 50 and peaking in 2021. This phase corresponds to intensified international collaboration, increased emphasis on evidence-based policy, and heightened societal concern with the psychological and social consequences of mass incarceration ( Kwiek, 2021).

*3.1.5 2021–2025: Sustained output with slight variability*

Although publication numbers remain high, slight fluctuations are evident, including a lower count in 2025 that likely reflects incomplete indexing rather than substantive decline. Overall, the temporal pattern demonstrates not merely quantitative growth but a qualitative shift toward institutionalization of resilience and motivation as core analytical constructs in correctional research.

Collectively, these trends suggest that the rise of this literature is not solely a function of technological change or publication volume, but reflects deeper transformations in penal philosophy, governance priorities, and scholarly orientation toward rehabilitation and reintegration ( Arbour et al., 2021). Understanding these temporal dynamics is essential for interpreting how conceptual frameworks and policy relevance co-evolve within the field.

### 3.2 Geographic distribution and collaboration network of authors

*3.2.1 Geographic distribution of authors*

Table 2 shows that research output is heavily concentrated in a small number of high-income countries, with the United States, Germany, and the United Kingdom accounting for a disproportionate share of institutional affiliations. Leading contributors are predominantly located in well-resourced academic and clinical centers, underscoring the role of funding availability, research infrastructure, and institutional capacity in shaping knowledge production ( Abdel-Salam & Kilmer, 2023).

**Table 2.** Geographic distribution of authors.

Affiliation	Count	*P*
United States	11	28.95
Germany	3	7.89
School of Engineering and Information Technology, University of New South Wales, Australian Defence Force Academy, Canberra, ACT, Australia	2	5.26
Safer Custody Group, HM Prison Service, United Kingdom	2	5.26
California State University, Fresno, United States	2	5.26
Bath Spa University, United Kingdom	2	5.26
Tambov State Technical University, Tambov, Russian Federation	2	5.26
United Kingdom	2	5.26
Department of Philosophy, Marquette University, Milwaukee, WI 53210-1881, PO Box 1881, United States	1	2.63
School of Psychology, University of Newcastle, NSW, Australia; National Drug Research Institute, Curtin University of Technology, WA, Australia; New South Wales Justice Health, NSW, Australia	1	2.63
Service de médecine interne, Maladies infectiousness, UCSA, Centre hospitalier régional Félix-Guyon, 97400 Saint-Denis, Reunion; UCSA de Lille-Loos-Sequedin, Centre hospitalier Régional de Lille, 59000 Lille, France; UCSA de Loos-Lez-Lille de Lille-Sequedin, UHSI, Centre hospitalier régional universitaire de Lille, 59000 Lille, France; Service de médecine polyvalente, Centre hospitalier régional Félix-Guyon, 97400 Saint-Denis, Reunion	1	2.63
Section of Trauma Studies, Division of Psychological Medicine and Psychiatry, Institute of Psychiatry, United Kingdom; Istanbul Center for Behavior Research and Therapy (ICBRT/DABATEM), Istanbul, Turkey	1	2.63
Florida State University, Tallahassee, FL, United States; Florida Department of Corrections, Tallahassee, FL, United States	1	2.63
Department of Psychology, Cardiff School of Health Sciences, University of Wales Institute Cardiff (UWIC), Llandaff Campus, CF52YB, Western Avenue, Cardiff, United Kingdom	1	2.63
Department of Psychology, University of Colorado at Boulder, United States; Center for AIDS Intervention Research (CAIR), Medical College of Wisconsin, United States; Department of Psychology, University of Nevada at Reno, United States; Department of Psychology, Center on Alcoholism, Substance Abuse, and Addictions (CASAA), University of New Mexico, United States	1	2.63
Treatment Research Institute, 600 Public Ledger Bldg., 150 South Independence Mall West, Philadelphia, PA 19106; Treatment Research Institute, University of Pennsylvania	1	2.63
Florida State University, College of Social Work, Tallahassee, FL 32306, 296 Champions Way, United States; University of Kansas, Lawrence, United States; University of Denver, Colorado, United States	1	2.63
Department of Psychiatry, University of New Mexico School of Medicine, Albuquerque, NM, United States; Correctional Medical Services, NM, United States; Santa Fe, NM 87505, 2442 Cerrillos Road #105, United States	1	2.63
University of Alabama at Birmingham, United States; Medical University of South Carolina, United States; Johns Hopkins University, United States	1	2.63
University of Antwerp, Faculty of Applied Economics, B-2000 Antwerpen, Prinsstraat 13, Belgium	1	2.63

Although emerging contributions from countries such as Russia, Belgium, Indonesia, Iran, and Turkey suggest a gradual diversification of the field, large regions — particularly Africa, Latin America, and parts of Asia — remain substantially underrepresented. This imbalance raises concerns about the cultural and institutional narrowness of dominant theoretical models and empirical findings.

**Figure 2.**
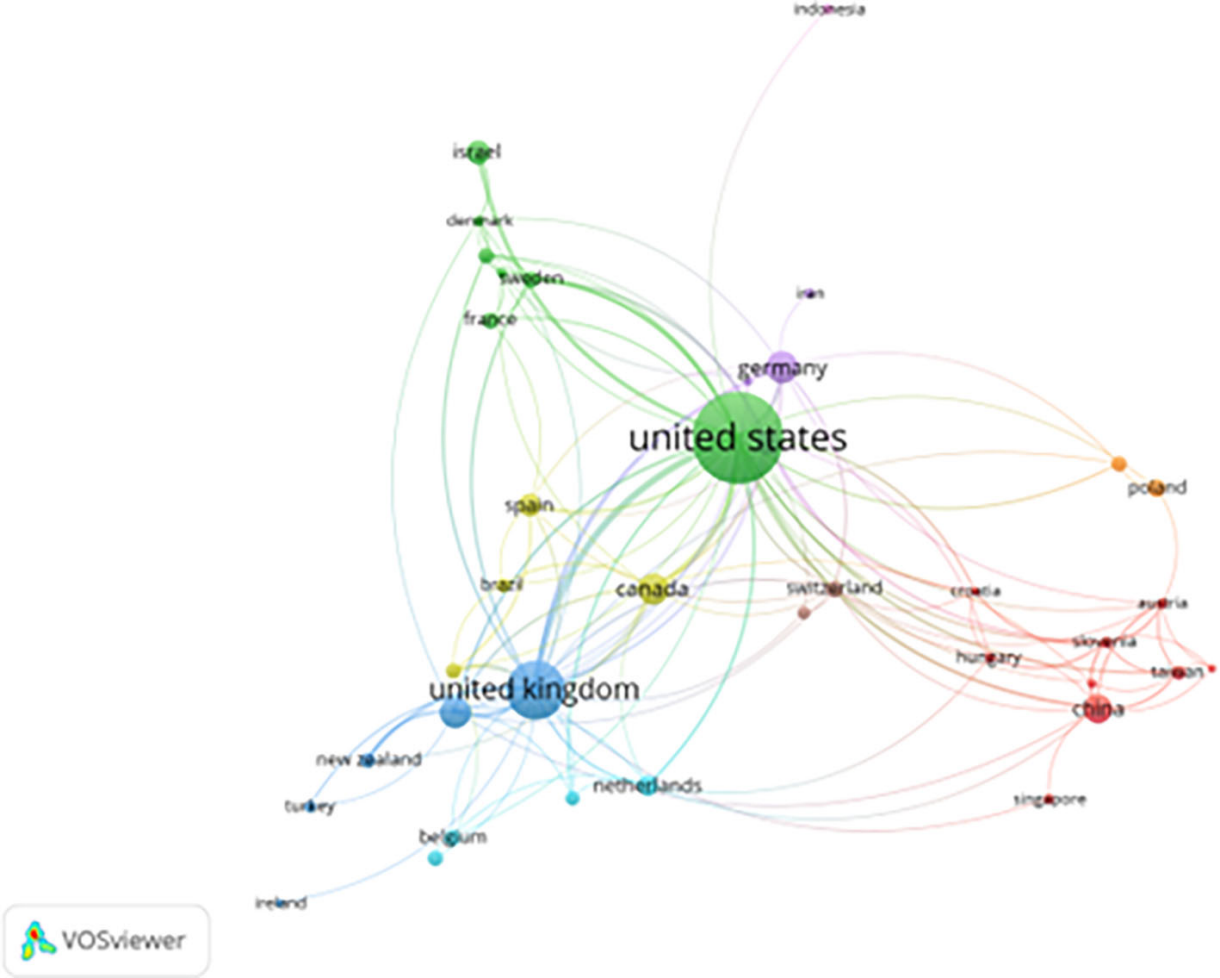
Country collaboration network in prisoner resilience and motivation research (VOSviewer visualization).

Figure 2 illustrates the structure of international collaboration networks. Strong ties among the United States, United Kingdom, Germany, Canada, and China indicate dense transnational research partnerships, supported by funding programs and shared policy agendas ( Batastini et al., 2024). European collaboration clusters further reflect coordinated research efforts linked to EU-level justice and rehabilitation initiatives.

In contrast, weaker integration of scholars from the Global South suggests persistent structural barriers including language dominance, uneven access to funding, and differences in correctional governance. These asymmetries shape not only who participates in research networks, but also which perspectives, problems, and populations become visible within the global literature.

*3.2.2 Collaboration networks among authors*

Table 3 identifies highly connected authors who function as central nodes within the co-authorship network. These collaborative hubs reflect stable research partnerships and thematic specialization, particularly in areas such as addiction recovery, therapeutic interventions, and motivational processes ( Mejia et al., 2021). While such clustering facilitates cumulative knowledge development, it may also contribute to intellectual path dependence, where dominant paradigms are reproduced through tightly interconnected scholarly communities.

**Table 3.** Collaboration networks among authors.

Author	Collaboration count	*P*
Stein L.A.R.	7	7.29
Sarchiapone M.	7	7.29
Ward T.	7	7.29
Roy A.	6	6.25
Boone C.	5	5.21
Moore J.L.	5	5.21
Brochu S.	5	5.21
Perc M.	5	5.21
Knight K.	5	5.21
Stuewig J.	4	4.17
Kiehl K.A.	4	4.17
Martin R.A.	4	4.17
Winder B.	4	4.17
Hoyt R.E.	4	4.17
Chen X.	4	4.17
Pinto da Costa M.	4	4.17
Carli V.	4	4.17
Day A.	4	4.17
Sánchez A.	4	4.17
Stams G.J.J.M.	4	4.17

The presence of fragmented or weakly connected segments suggests that early-career researchers and scholars from underrepresented regions may face limited access to influential networks. This has implications for diversity of theoretical innovation, methodological pluralism, and cross-cultural sensitivity within the field.

**Figure 3.**
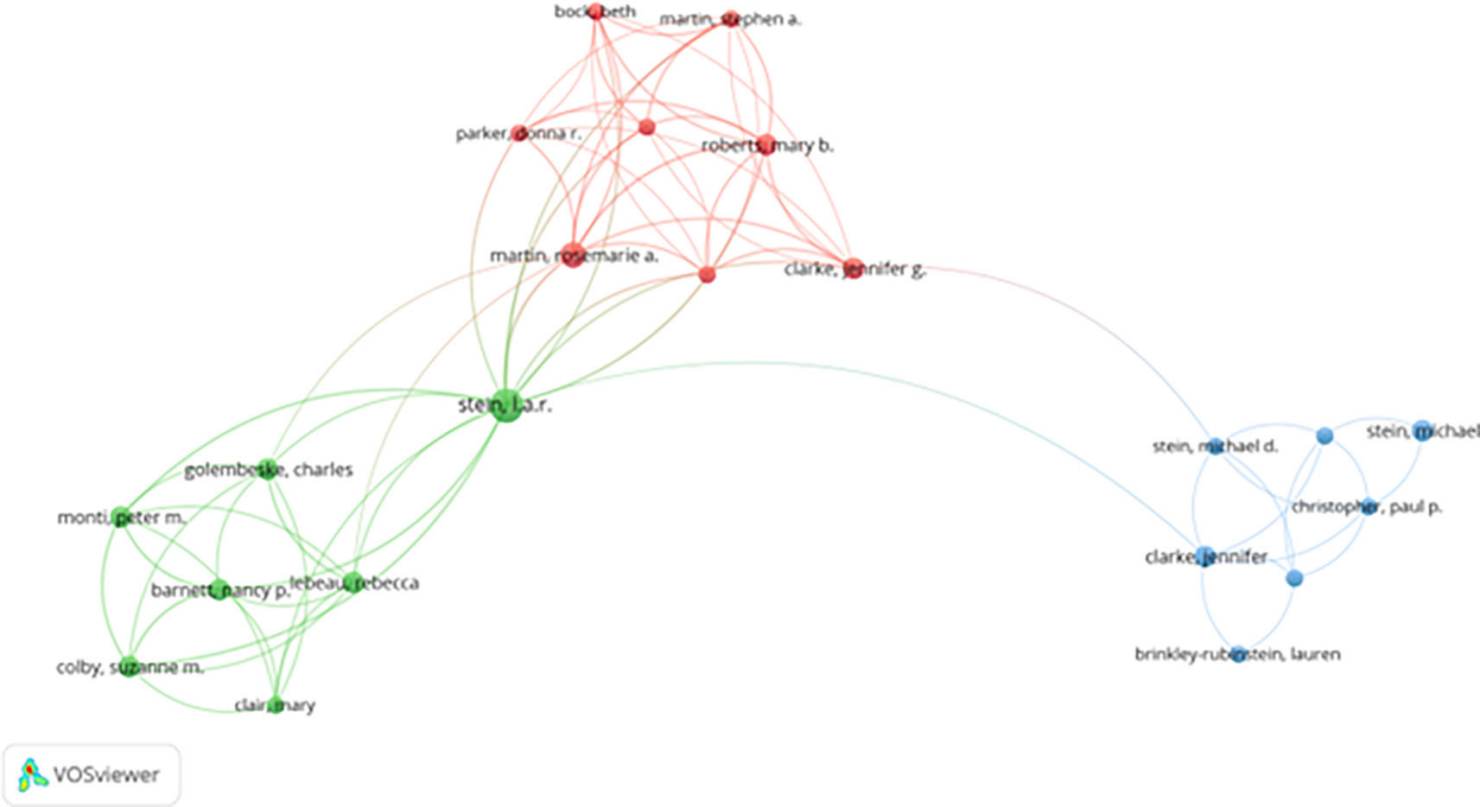
VosViewer analysis on author co-authorship network in prisoner resilience and motivation research.

Figure 3 (co-authorship network via VOSviewer) illustrates these clusters, with several prominent collaborative hubs centered around authors like Stein and Martin. These clusters show thematic specialization, such as drug addiction recovery, psychological therapy, and motivational enhancement in correctional settings.

Despite strong internal collaboration, limited cross-cluster integration suggests missed opportunities for broader theoretical exchange. Strengthening interdisciplinary and cross-regional collaboration could foster innovation and methodological diversity in prisoner rehabilitation research.

*3.2.3 Institutional contributions and collaborations*

**Figure 4.**
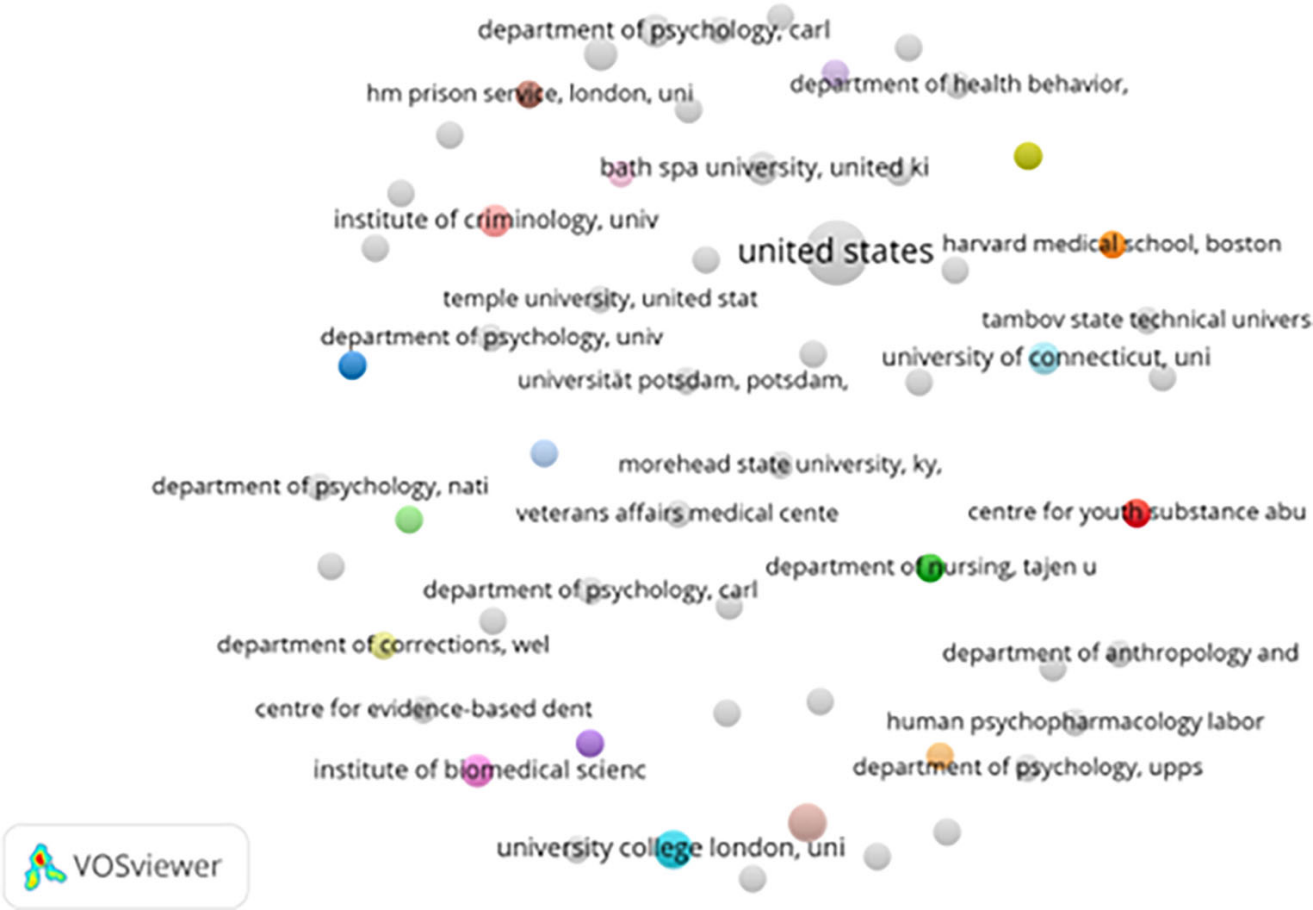
VosViewer analysis on institutional collaboration network in prisoner resilience and motivation research.

Figure 4 demonstrates that institutional collaboration is similarly concentrated among elite universities, medical schools, and government-linked research centers, primarily in North America and Europe. These institutions often serve as bridges between academic research and policy implementation, facilitating translation of evidence into correctional practice ( Hedges et al., 2021).

However, the marginal participation of institutions from low- and middle-income countries limits the global applicability of prevailing models of rehabilitation, resilience, and motivation. Without broader institutional inclusion, the field risks reproducing a narrow set of assumptions about incarceration, rehabilitation, and social reintegration that may not align with diverse cultural, legal, and economic contexts.

Overall, Section 3.2 highlights that while the field is expanding and increasingly collaborative, it remains structurally unequal. Addressing these asymmetries is not only a matter of representational equity but is essential for developing theoretically robust, culturally grounded, and globally relevant knowledge about resilience and motivation in correctional settings.

### 3.3 Thematic areas and research focus

*3.3.1 All keywords*

**Figure 5.**
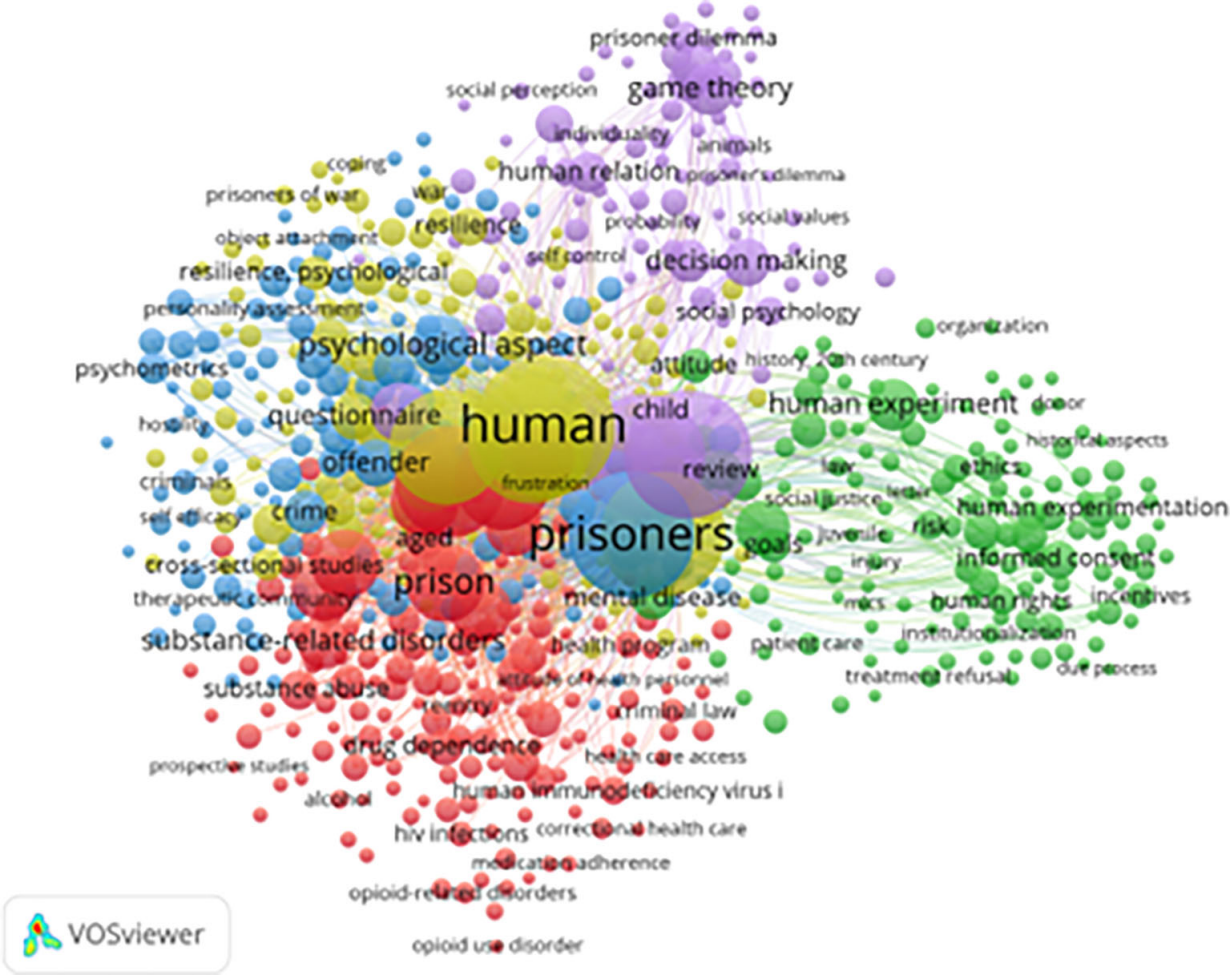
VosViewer analysis on keyword co-occurrence network in resilience and motivation research. Note: Minimum number of occurrences of keyword 7 of the 6221 keywords, 650 meet the thresholds.

Figure 5 presents a keyword co-occurrence network generated using VOSviewer to visualize dominant themes and conceptual linkages in research on resilience and motivation among prisoners. A total of 650 keywords met the minimum inclusion threshold (out of 6,221 with at least seven occurrences), providing a structured representation of the field’s intellectual landscape.

The most frequently co-occurring terms include *prisoners*, *resilience*, *motivation*, *coping*, *psychological aspects*, *self-efficacy*, *drug addiction*, and *rehabilitation*. The density and strength of links among these terms indicate that the field is inherently interdisciplinary, integrating perspectives from correctional psychology, criminology, public health, addiction studies, and behavioral science ( Radhakrishnan et al., 2017; Bukar et al., 2023). This pattern reflects a shift away from isolated disciplinary silos toward a more integrated conceptualization of incarceration as simultaneously a psychological, social, health, and institutional phenomenon.

Four major thematic clusters emerge from the network.

The Blue Cluster (Psychosocial Resilience) centers on emotional regulation, coping, self-efficacy, and decision-making. This cluster captures research that conceptualizes resilience as an adaptive psychological process enabling incarcerated individuals to manage stress, maintain emotional stability, and navigate institutional constraints. Its prominence suggests that resilience is primarily framed in individual-level psychological terms, even when embedded in broader institutional contexts.

The Red Cluster (Substance Abuse and Rehabilitation) includes terms such as *addiction*, *opioid use disorder*, and *dependence*, highlighting the strong empirical and policy-driven focus on substance use as both a contributor to incarceration and a target of rehabilitation ( Schulz et al., 2023). This cluster reflects the central role of therapeutic interventions in contemporary correctional research and underscores the convergence of correctional and public health agendas.

The Green Cluster (Legal and Ethical Issues) encompasses keywords such as *criminal law*, *human rights*, *informed consent*, and *institutionalization*, indicating that resilience and motivation research is situated within normative and regulatory debates about punishment, rights, and the ethical governance of incarcerated populations. This cluster reflects the increasing influence of rights-based and reform-oriented perspectives in the field.

The Yellow Cluster (Behavioral Decision-Making) features terms such as *game theory*, *prisoner’s dilemma*, and *decision-making*, signaling the growing application of behavioral economics and social psychology to understand how individuals make choices under conditions of coercion, scarcity, and uncertainty.

Additional terms such as *HIV prevention*, *treatment adherence*, and *healthcare access* highlight increasing attention to health inequities in correctional settings, while the appearance of *women prisoners* and *gender* indicates an emerging, though still limited, recognition of heterogeneity within incarcerated populations.

Despite this conceptual diversity, notable gaps persist. Keywords related to vocational training, post-incarceration employment, and economic reintegration remain underrepresented, despite their central importance for long-term desistance and social stability. Similarly, limited emphasis on cultural adaptation and ethnic diversity suggests that much of the literature continues to rely on Western institutional and cultural assumptions, potentially constraining the global relevance of dominant models.

Overall, the keyword network reveals a clear transition from punitive and deterrence-oriented frameworks toward rehabilitative, rights-based, and psychosocial approaches. At the same time, it highlights the need for greater integration of structural, cultural, and post-release dimensions into future research in order to develop more comprehensive and context-sensitive understandings of resilience and motivation in correctional settings.

*3.3.2 Keyword co-occurrence and emerging trends*

**Figure 6.**
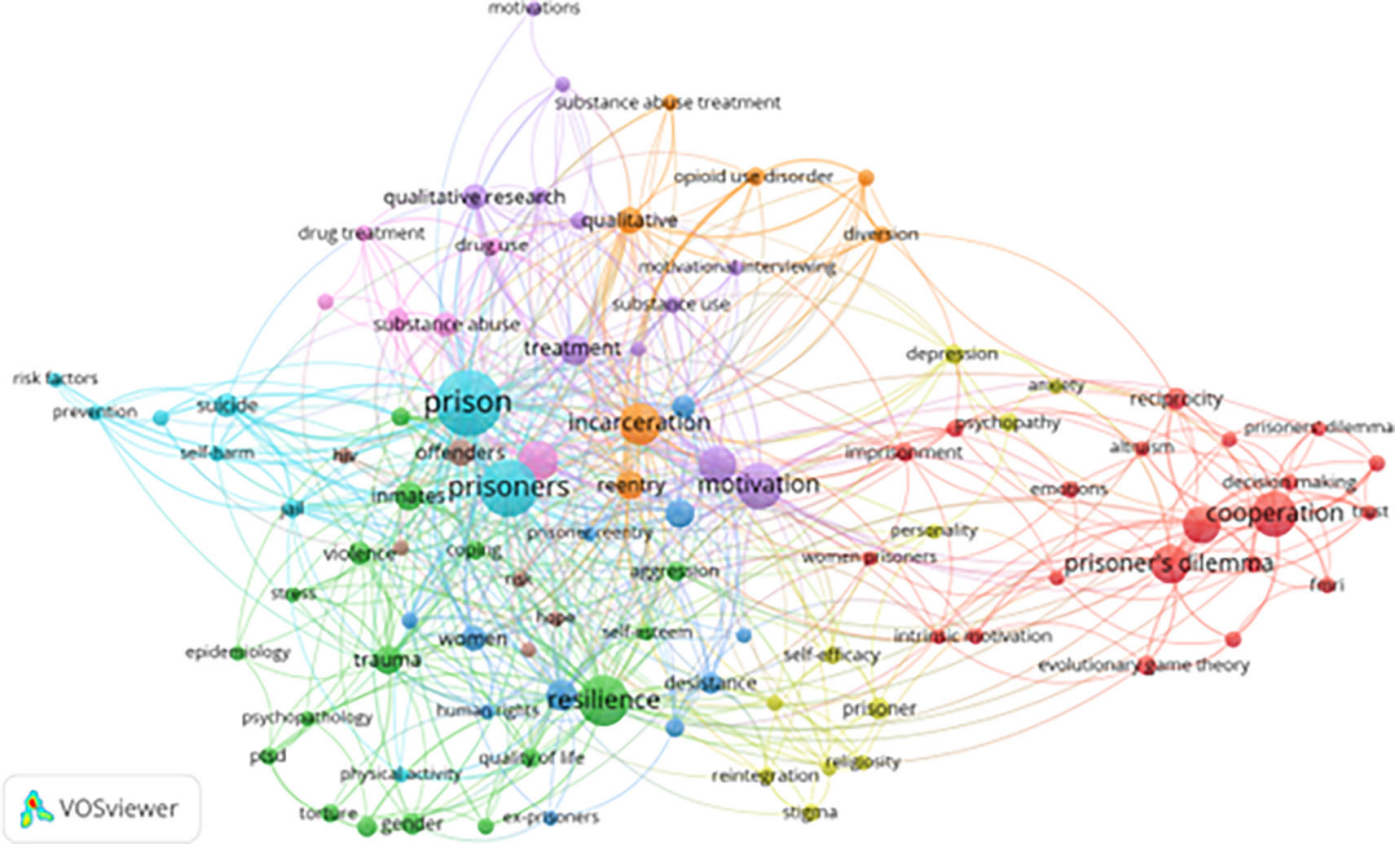
VosViewer analysis on keyword co-occurrence and emerging trends. Note: Minimum number of occurrences of a keyword 5 of the 2678 keywords, 99 meet the thresholds.

Figure 6 presents a temporal keyword co-occurrence network based on 99 keywords (from a total of 2,678 with at least five occurrences), allowing examination of both established thematic domains and emerging areas of scholarly attention.

Several prominent and evolving clusters are evident.

The Psychological Resilience and Coping Strategies cluster is dominated by keywords such as *trauma*, *self-esteem*, *quality of life*, and *human rights*. This cluster reflects a growing emphasis on trauma-informed, rights-based, and well-being-oriented approaches to incarceration, as well as increasing attention to subgroup-specific resilience strategies, particularly among women and minority populations. This shift indicates movement away from deficit-based models toward frameworks that emphasize dignity, psychological safety, and personal growth.

The Substance Abuse Recovery cluster includes terms such as *opioid use disorder*, *pharmacotherapy*, and *motivational interviewing*, underscoring the continued centrality of addiction treatment in correctional research and practice. This cluster is closely linked to motivational theories and cognitive-behavioral recovery models, particularly within therapeutic community approaches that emphasize structured peer support and motivational change ( Sacks et al., 2012). The persistence of this cluster highlights the enduring intersection between criminal justice and public health.

The Post-Incarceration Reintegration cluster features keywords such as stigma, hope, goal setting, and faith-based rehabilitation, reflecting growing scholarly concern with the social, psychological, and moral dimensions of reentry. This trend suggests increasing recognition that rehabilitation does not end at the prison gate, and that successful reintegration depends on both individual agency and community-level support structures.

This moral and affective dimension of reintegration is further elaborated by Shield (2025), who argues that emotions such as shame should be understood as relational and psychological processes rather than ontological conditions, and that addressing shame is crucial for sustaining hope, motivation, and long-term reintegration trajectories among justice-involved populations.

The Decision-Making Models cluster encompasses terms such as *behavioral economics*, *trust*, *risk-taking*, and *prisoner’s dilemma*, indicating the integration of formal decision theory and behavioral science into the study of inmate behavior. This work reflects a more nuanced understanding of how incarcerated individuals navigate social interactions, institutional incentives, and risk under conditions of constraint.

Finally, the Ethical and Legal Concerns cluster includes terms such as *medical ethics*, *criminal justice reform*, and *prison health disparities*, signaling a growing critical orientation toward systemic injustice, institutional harm, and the moral legitimacy of contemporary penal practices. This cluster reflects the increasing influence of normative and policy-oriented scholarship in shaping correctional research agendas.

Collectively, these emerging trends indicate a broader paradigmatic shift in correctional research—from models centered on institutional control, risk management, and punishment toward frameworks emphasizing rehabilitation, resilience, human rights, and restorative justice. However, the network also reveals persistent blind spots. Cultural adaptation of interventions, technological innovation in rehabilitative practices, and the long-term socioeconomic trajectories of formerly incarcerated individuals remain underexplored, limiting the field’s capacity to generate context-sensitive and sustainable solutions.

Overall, the temporal keyword analysis demonstrates both the maturation and the partial reorientation of the field, while simultaneously identifying critical areas for future research that are necessary to support equitable, effective, and globally relevant correctional rehabilitation strategies.

*3.3.3 Index keywords*

Index keywords refer to standardized subject terms assigned by indexing services (e.g., Scopus and journal metadata systems) to represent the primary conceptual content of each publication. Unlike author-provided keywords, which reflect authors’ self-descriptions of their work, index keywords offer a normalized vocabulary that enables systematic classification, cross-study comparison, and bibliometric mapping of research domains.

**Figure 7.**
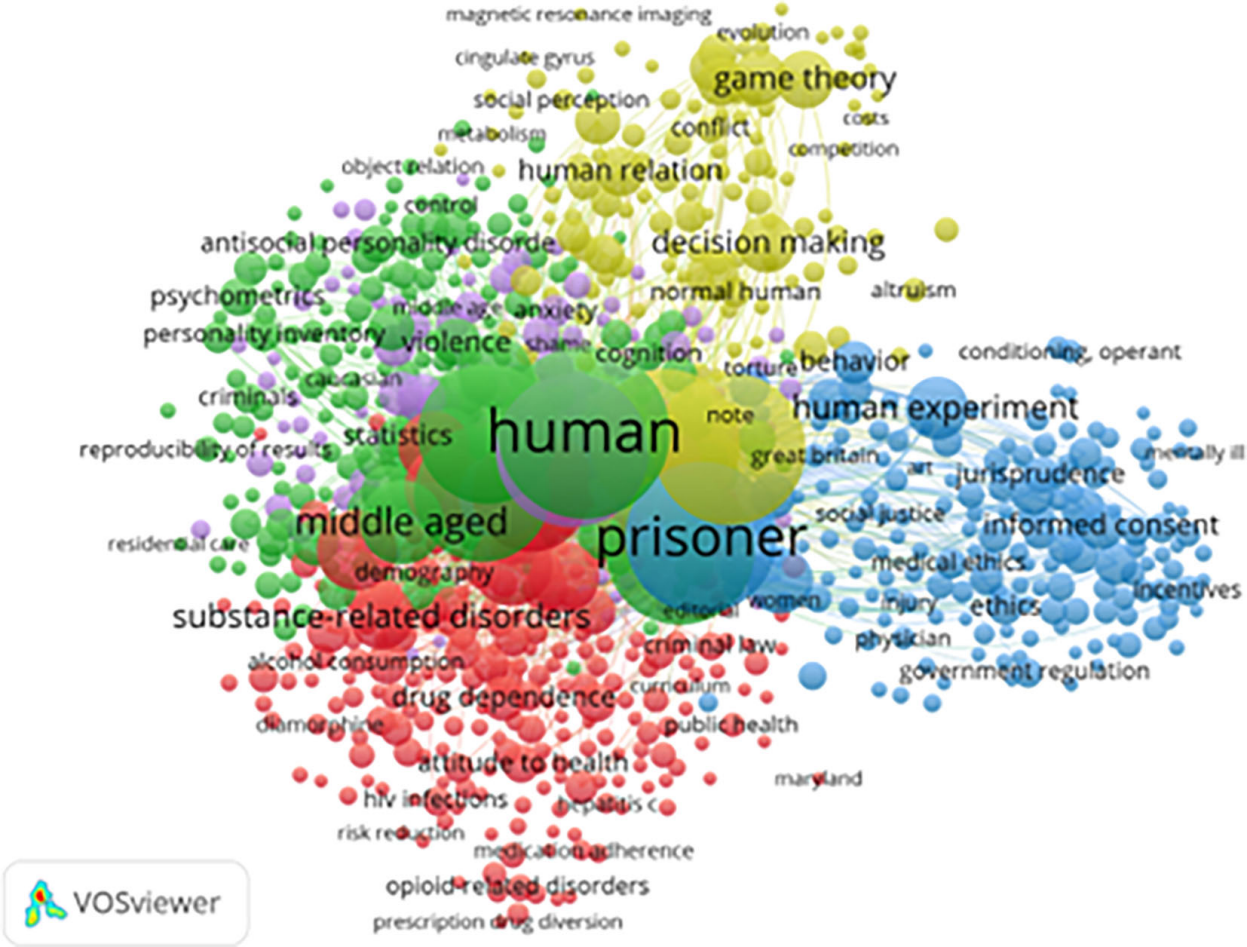
VosViewer analysis on index keywords in resilience and motivation research among prisoners. Note: Minimum number of occurrences of a keyword 5 of the 4203 keywords, 824 meet the thresholds.

Figure 7 presents the index keyword clusters used to classify core topics in the field. A total of 824 index keywords (from 4,203 with at least five occurrences) met the threshold for inclusion. These clusters both corroborate and refine the thematic patterns identified in earlier keyword analyses.

The Green Cluster (Psychological Themes) includes terms such as anxiety, personality, and psychometrics, indicating sustained scholarly attention to how individual psychological traits, emotional regulation, and measurement frameworks relate to resilience and behavioral adaptation in prison settings. This cluster reflects the continued dominance of psychological assessment and trait-based approaches within correctional research.

The Red Cluster (Health and Addiction) is dominated by terms such as drug use, alcohol, medication adherence, and HIV, highlighting the centrality of substance use disorders and health vulnerabilities within incarcerated populations. This cluster underscores the close empirical and institutional link between correctional research and public health, particularly in relation to harm reduction, treatment compliance, and health-related rehabilitation needs.

The Blue Cluster (Legal and Ethical Dimensions) includes terms such as jurisprudence, informed consent, and government regulation, reflecting the legal and normative frameworks that structure research, intervention, and governance in correctional settings. This cluster indicates that resilience and motivation research is not purely psychological but is embedded within regulatory, ethical, and rights-based discourses concerning incarceration.

The Yellow Cluster (Decision-Making and Institutional Behavior) encompasses terms such as game theory, cooperation, cognitive processing, and trust, pointing to a growing interest in how incarcerated individuals make decisions, form social relationships, and navigate institutional power structures. This cluster reflects the integration of behavioral economics, social psychology, and institutional theory into the study of prison life.

Despite this conceptual breadth, several important gaps remain evident in the index keyword structure. First, gender-specific interventions remain underrepresented, with relatively limited attention to women, LGBTQ+ individuals, and elderly prisoners, despite their distinct vulnerabilities and rehabilitative needs. Second, cross-cultural resilience frameworks are rare, reflecting the continued dominance of Western-centric theoretical models and limiting the applicability of findings across diverse correctional systems. Third, post-release economic reintegration, including employment readiness and labor market access, appears infrequently despite its central importance for long-term desistance and social reintegration ( Chandrasekaran et al., 2024).

Taking together, the index keyword analysis indicates that while the field has diversified and matured, it remains uneven in its thematic, demographic, and geographic coverage. Advancing research on resilience and motivation among incarcerated populations will therefore require greater attention to cultural context, gender and age diversity, and the structural conditions shaping reintegration outcomes, alongside sustained interdisciplinary collaboration among psychology, criminology, public health, law, and social policy.

*3.3.4 Temporal and thematic evolution of research*

**Figure 8.**
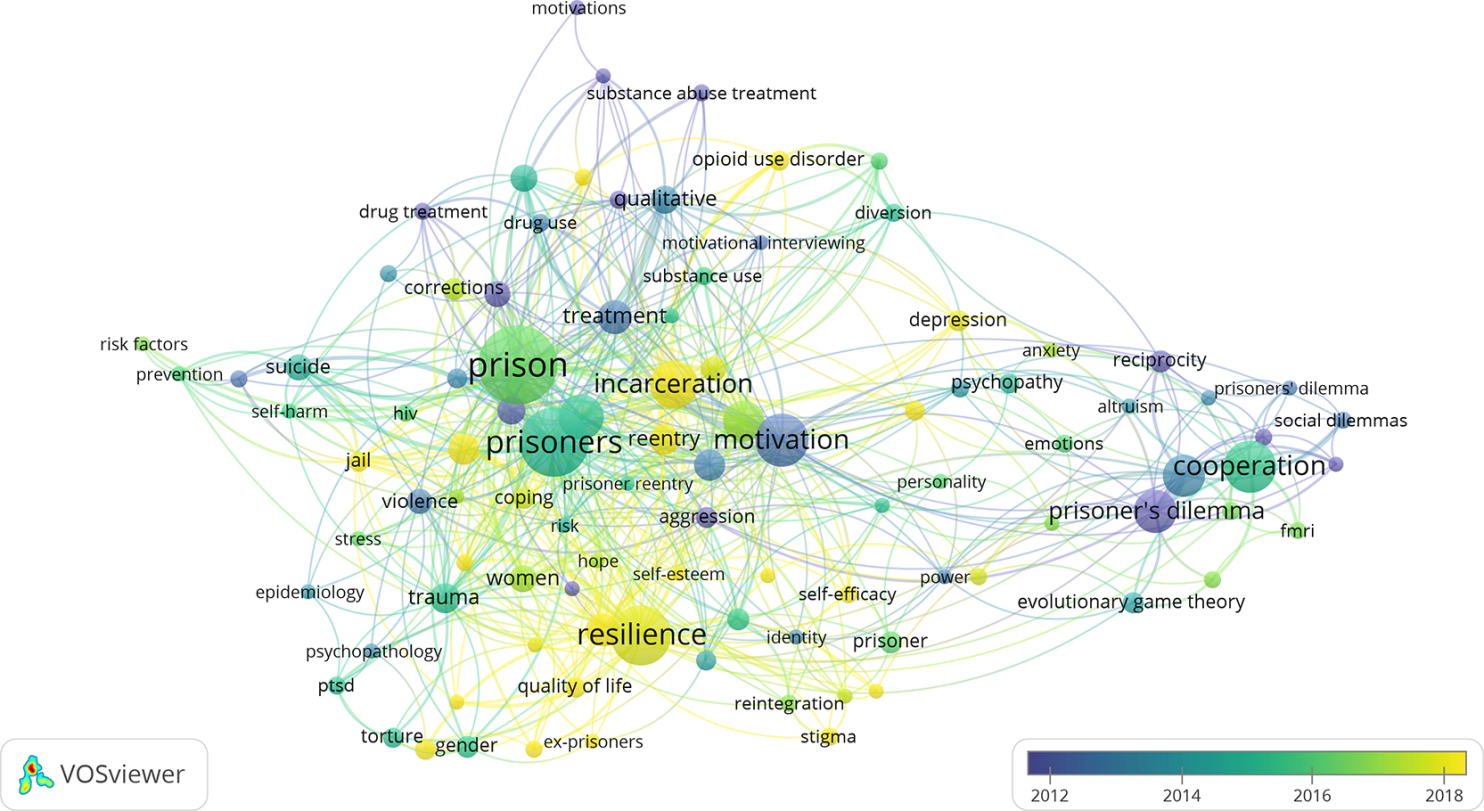
Temporal evolution of research themes (1912–2024).

The temporal overlay visualization ( Figure 8) illustrates a clear chronological shift in the conceptual focus of research on prisoners’ resilience and motivation. Early studies, represented by cooler colors, are predominantly oriented toward psychological adaptation, coping, and adjustment to institutional stress. These early themes reflect a primarily clinical and individual level understanding of incarceration as a psychological stressor requiring symptom management and behavioral stabilization.

Over time, warmer color transitions indicate a gradual expansion toward motivation, rehabilitation, reintegration, and rights-based frameworks. This progression signals an epistemic shift from viewing incarcerated individuals primarily as objects of control and treatment toward recognizing them as agents capable of growth, learning, and social reintegration. The increasing convergence between resilience, motivation, and rehabilitation domains reflects the emergence of a more holistic correctional paradigm that integrates psychological adaptation with social, moral, and institutional dimensions of change.

The overlay also reveals that newer themes tend to cluster around concepts related to identity, agency, and future orientation, such as goal setting, hope, and reintegration. This indicates a move away from short-term symptom reduction toward longer-term developmental and life-course perspectives on incarceration and desistance. Such a transition aligns with broader shifts in criminological theory and penal policy emphasizing strengths-based, restorative, and community-oriented approaches.

In parallel, co-authorship mapping demonstrates that scholarly collaboration has become increasingly international since approximately 2015, particularly among researchers based in the United Kingdom, the United States, Australia, Malaysia, and South Africa. This growing interconnectedness suggests not only greater technical capacity for collaboration but also expanding theoretical exchange across legal systems, cultural contexts, and disciplinary traditions. The globalization of research networks mirrors the broadening theoretical lens through which resilience and motivation among incarcerated individuals are now conceptualized.

However, this temporal and geographic expansion remains uneven. While international collaboration has increased, it continues to be dominated by institutions in high-income, English-speaking countries, and scholars from many regions remain marginally connected. As a result, the apparent diversification of themes may still be constrained by a relatively narrow epistemic base. Without broader inclusion of perspectives from underrepresented regions and alternative penal traditions, the field risks reproducing a limited set of assumptions about rehabilitation, agency, and change.

Overall, the temporal evolution of themes reflects both conceptual maturation and normative reorientation within the field, from control-oriented and deficit-based models toward frameworks emphasizing resilience, motivation, dignity, and reintegration. At the same time, it highlights the need for continued critical reflection on whose knowledge shapes this evolution and how global inequalities in research capacity influence the direction and content of scholarly inquiry.

Overlay visualization map generated using VOSviewer (version 1.6.19) based on keyword co-occurrence analysis. The color gradient represents the average publication year, showing the chronological progression of key research themes from early psychological adaptation studies (blue) to more recent emphases on motivation, resilience, and reintegration (yellow). Larger nodes indicate higher keyword frequency, while line thickness represents the strength of co-occurrence links. The map highlights the temporal convergence between resilience, motivation, and rehabilitation research, reflecting the field’s increasing interdisciplinarity and theoretical diversification.

### 3.4 Citation analysis and research influence

**Figure 9.**
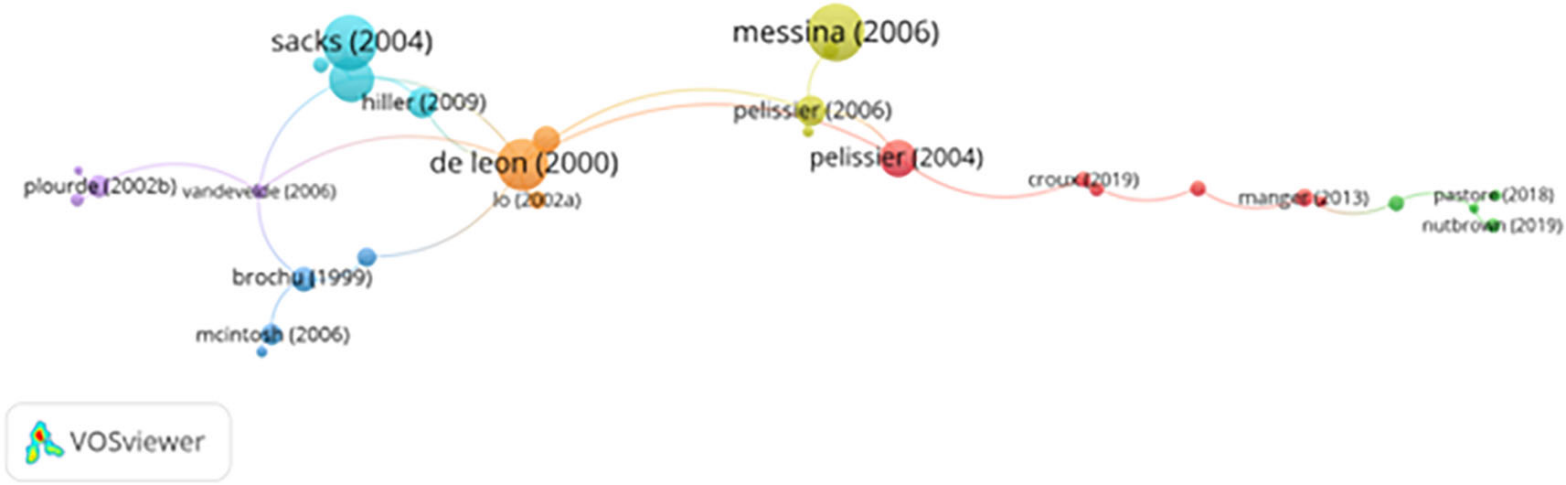
VosViewer analysis on citation analysis and research influence. Note: Minimum number of citations of a document: 3 of the 1309 documents, 907 meet the thresholds.

Figure 9 presents the citation network of key publications in prisoner resilience and motivation research. Central nodes represent highly cited works, while node size and link thickness reflect citation volume and relational influence within the scholarly network.

Several foundational works form the core of this network, including De Leon (2000), Peters et al. (2004), Messina et al. (2006), Manger et al. (2010) and Borseth et al. (2025). These studies established the empirical and theoretical foundations of the field, particularly in relation to therapeutic communities, addiction treatment, educational engagement, and the role of motivation in rehabilitation. Subsequent contributions by Pelissier et al. (2003) and Messina et al. (2006) further demonstrated how structured educational and therapeutic interventions enhance intrinsic motivation and reduce recidivism through evidence-based rehabilitative frameworks.

Together, these influential works contributed to a paradigmatic shift from deterrence-oriented correctional models toward rehabilitative, motivational, and psychologically informed approaches, supported by cognitive-behavioral principles ( Christensen & Hesstvedt, 2024). More recent citation activity reflects an expanding focus on post-release trajectories and reintegration processes. Studies by King & Smith (2024), Sahoo et al., (2025), Chouhy et al. (2020) and Link et al. (2019) emphasize how employment access, social support, and mental health services shape long-term resilience, desistance, and social inclusion. This shift signals a maturation of the field toward life-course, systems-oriented, and interdisciplinary perspectives.

*3.4.1 Gaps and structural asymmetries*

Despite these advances, the citation network reveals persistent structural and epistemic imbalances.

First, non-Western scholarship remains underrepresented. Highly cited works are overwhelmingly produced by institutions in North America and Western Europe, reflecting historical dominance in criminological theory production and unequal global research capacity. As a result, core concepts such as rehabilitation, desistance, and reentry are largely framed through Western penal and cultural assumptions.

Second, there is a lack of comparative cross-cultural analysis. Few influential studies examine how resilience and motivation operate within diverse legal systems, religious traditions, and socio-economic contexts. Incorporating perspectives from Asia, Africa, Latin America, and the Middle East would strengthen the field’s explanatory depth and policy relevance, particularly in settings where incarceration intersects with poverty, community justice practices, and informal social regulation.

Third, epistemic hierarchies in citation practices persist. Citation visibility is shaped by journal indexing systems and linguistic accessibility, privileging English-language publications and marginalizing regionally focused or non-indexed scholarship. As Bosworth (2023) argues, this dynamic reproduces epistemic coloniality by positioning Global North frameworks as universal standards.

**Journal co-citation patterns and interdisciplinary convergence**

**Figure 10.**
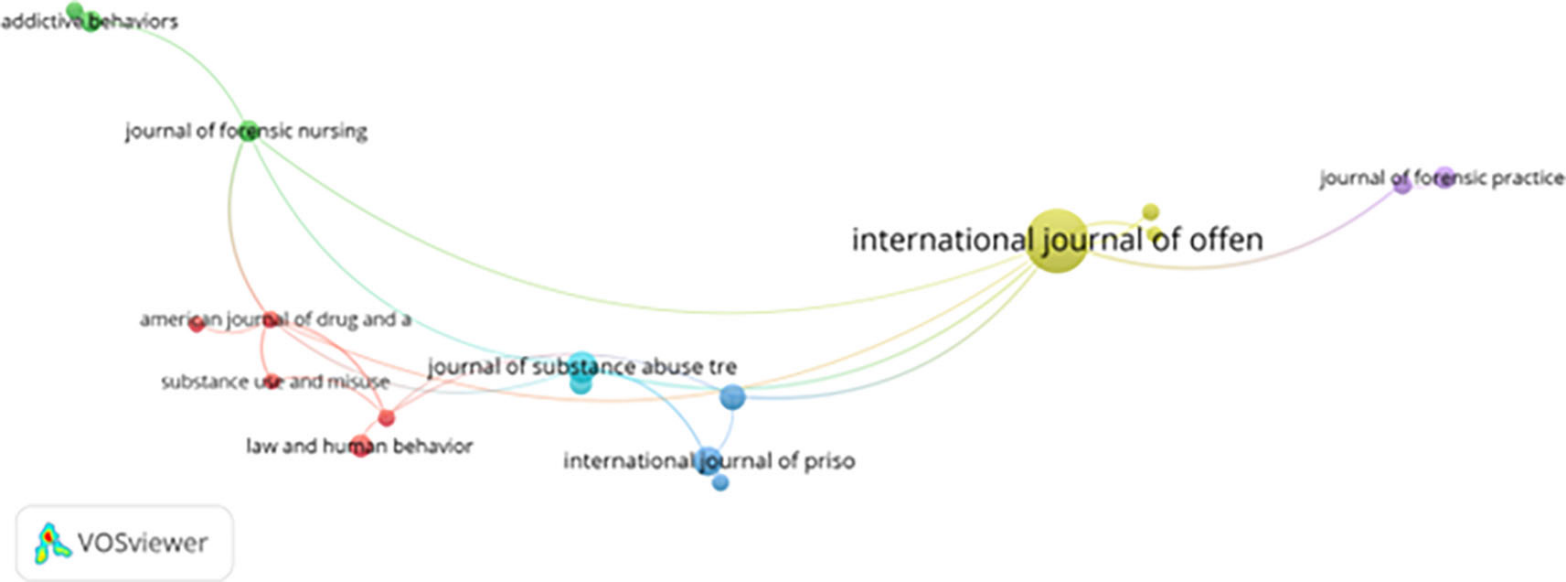
VosViewer analysis on journal co-citation analysis of the most prominent academic sources. Note: Minimum number of documents of a source 5 of the 784 sources, 32 meet the thresholds.

Figure 10 displays journal co-citation patterns, highlighting the interdisciplinary foundations of the field. Journals such as the *Journal of Offender Therapy and Comparative Criminology*, *Addictive Behaviors*, *American Journal of Drug and Alcohol Abuse*, and *Law and Human Behavior* function as central nodes, reflecting convergence across addiction research, forensic psychology, behavioral science, and criminal justice reform.

This transdisciplinary integration strengthens theoretical robustness and methodological diversity. However, the limited representation of journals from the Global South restricts the circulation of locally grounded knowledge and may constrain innovation in culturally responsive correctional practice.

*3.4.2 Interpretive and sociopolitical reflection*

The concentration of citations within Western academic systems reflects not only material inequalities in funding and infrastructure but also deeper ideological continuities rooted in the postcolonial history of penal governance. Criminological theories developed in the Global North have often been transferred to other regions without sufficient cultural translation, reinforcing narrow models of rehabilitation that prioritize institutional control over community-based and culturally embedded forms of transformation ( Carrington et al., 2018).

Drawing on Braithwaite’s (2002) concept of responsive regulation and Garland’s (2018) analysis of penal modernity, it becomes evident that global diffusion of correctional knowledge entails moral, political, and cultural negotiation. In many non-Western contexts, resilience and motivation are intertwined with religious belief systems, collective identity, and familial obligation — dimensions that remain underrepresented in mainstream literature. Recognizing these pluralities is essential for developing a decolonized and context-sensitive criminology.

*3.4.3 Synthesis and practical relevance*

In summary, citation and co-citation analyses reveal the evolution of the field from early emphases on therapeutic communities and addiction recovery to contemporary concerns with reintegration, mental health, and justice reform. While the field is empirically rich and theoretically sophisticated, its future development depends on structural inclusivity, cultural reflexivity, and democratization of knowledge production.

Promoting South–South research collaboration, expanding open-access bibliometric resources, and diversifying editorial and peer-review representation would help redress existing imbalances. Such efforts would foster a more equitable global exchange of ideas and support the development of rehabilitation frameworks that reflect the lived realities of incarcerated populations across diverse cultural and institutional contexts.

## 4. Conclusions

This bibliometric review provides a comprehensive mapping of more than a century of scholarship (1912–2024) on resilience and motivation among incarcerated populations. By integrating temporal trends, thematic structures, citation networks, and collaboration patterns, the analysis demonstrates that this field has evolved from a narrow focus on psychological adjustment and behavioral control into a multidimensional, interdisciplinary domain encompassing rehabilitation, reintegration, public health, legal reform, and social inclusion.

The findings reveal that resilience and motivation have increasingly been conceptualized not as isolated psychological traits, but as dynamic, contextually embedded processes shaped by institutional environments, social relationships, and structural conditions. This conceptual shift reflects a broader transformation in correctional research and policy—from punitive and risk-oriented models toward frameworks emphasizing personal agency, dignity, recovery, and social reintegration.

At the same time, the analysis identifies persistent structural and epistemic asymmetries. Knowledge production remains heavily concentrated in Western, high-income countries, and dominant theoretical frameworks continue to reflect specific legal, cultural, and institutional assumptions. As a result, much of the global diversity in correctional systems, cultural understandings of moral transformation, and community-based forms of rehabilitation remains underrepresented in the literature. This imbalance limits both the explanatory power and the practical relevance of existing research for non-Western contexts.

From a theoretical perspective, this review underscores the need to integrate psychological, sociological, legal, and cultural approaches to better understand how resilience and motivation operate under conditions of confinement and transition. Future research should move beyond individual-level models to incorporate institutional design, staff-prisoner relations, community structures, labor markets, and post-release support systems as constitutive elements of rehabilitative processes.

From a methodological standpoint, the findings highlight the value of bibliometric approaches for identifying intellectual trajectories, structural gaps, and emerging paradigms within complex interdisciplinary fields. However, bibliometric visibility should not be conflated with epistemic validity. Greater attention to multilingual, regional, and non-indexed scholarship is essential for constructing a more inclusive and globally relevant knowledge base.

From a policy and practice perspective, the results suggest that effective correctional rehabilitation depends not only on individual psychological change but also on the availability of supportive institutional and social environments that enable sustained motivation and resilience beyond incarceration. Interventions grounded in dignity, autonomy, social connection, and cultural meaning are more likely to produce durable outcomes than those focused solely on compliance or risk reduction.

In conclusion, research on prisoner resilience and motivation stands at a critical juncture. Its future development will depend on the field’s capacity to embrace theoretical pluralism, cultural reflexivity, and structural inclusivity. By expanding its epistemic boundaries and deepening its engagement with underrepresented contexts, this scholarship can contribute not only to improved correctional outcomes, but also to a more humane, equitable, and context-sensitive vision of justice and social repair.

## Ethical considerations

Not applicable.

## Data Availability

The dataset generated and analysed during the current study is openly available at: https://doi.org/10.6084/m9.figshare.29321222.v1 [ Hussin (2025)]. This project contains following datafiles:
1. scopus (2).csv 2. PRISMA checklist Data are available under the terms of the Creative Commons Zero “No rights reserved” data waiver (CC0 1.0 Public domain dedication).
