# Supplementary material for: A bibliometric review of research on resilience, motivation and prisoners, 1912-2024
Source: F1000Res. 2026 Jan 6;14:760. Originally published 2025 Aug 1. [Version 3] doi: 10.12688/f1000research.164019.3 (PMC12789858; doi:10.12688/f1000research.164019.3)
Supplement: Peer review history

# Reviewer report: Reviewer response for version 3

**DOI:** 10.5256/f1000research.194521.r448183

**Published:** 2026-01-10

**Authors:** Milena Milićević, Svetlana Pavlović

**Affiliations:** 1 Institute of Criminological and Sociological Research, Belgrade, Serbia

Dear Authors,

 Dear Editors,

 We have reviewed the revised manuscript and are glad to see that our earlier comments have been addressed. The changes have improved the paper’s conceptual framing, methods, and analysis.

 The manuscript now clearly explains how resilience and motivation are connected in correctional settings. It provides a clearer rationale for using the bibliometric approach, and the flow and organisation of the literature review and methods sections are clearer, as are the tables and terms that were previously unclear.

 The Results and Discussion sections are now more analytical and better connected to the study’s institutional and theoretical implications. This makes the study more useful for prison and rehabilitation research.

 Any remaining issues are minor and can be addressed during copyediting if needed.

 We recommend accepting the manuscript.

Are the rationale for, and objectives of, the Systematic Review clearly stated?

Yes

Is the statistical analysis and its interpretation appropriate?

Yes

If this is a Living Systematic Review, is the ‘living’ method appropriate and is the search schedule clearly defined and justified? (‘Living Systematic Review’ or a variation of this term should be included in the title.)

Not applicable

Are sufficient details of the methods and analysis provided to allow replication by others?

Yes

Are the conclusions drawn adequately supported by the results presented in the review?

Yes

Reviewer Expertise:

prison research

We confirm that we have read this submission and believe that we have an appropriate level of expertise to confirm that it is of an acceptable scientific standard.

# Reviewer report: Reviewer response for version 2

**DOI:** 10.5256/f1000research.189413.r440283

**Published:** 2025-12-30

**Authors:** Raechel N. Soicher

**Affiliations:** 1 Teaching + Learning Lab, Massachusetts Institute of Technology, Cambridge, MA, USA

Thank you for the opportunity to review Version 2 of “A bibliometric review of research on resilience, motivation and prisoners, 1912-2024” for *F1000 Research*. This article reports on the bibliometric analysis of published research on the topics of resilience and motivation in incarcerated populations. While I think this type of analysis is incredibly important and will be a meaningful contribution to the field, I have some major concerns about the robustness of the research as it is currently described.

**Major Issues**

- **Meta-Analysis**
  - In my discipline, a quantitative meta-analysis refers specifically to “a powerful statistical method that combines numerical results from multiple independent studies (like randomized trials or surveys) to produce a single, more precise estimate of an effect.” I do not see a meta-analysis of this kind anywhere in the article.
- **Methodology**
  - The appearance of “prisoner’s dilemma” in Figure 8 has raised red flags for me about the overall accuracy of the inclusion criteria and has the potential to invalidate any of the findings related to decision-making. The “Prisoner’s Dilemma” is the name given to any task used in decision-making research (most notably in cognitive psychology and economics) where cooperation between two players will have a higher payoff than either individual payoff. Notably, this task is commonly used in research **NOT involving incarcerated populations at all**. There are several examples of articles of this nature in the published dataset, but since there is no indication of whether each article was included, it is impossible to tell whether the final analysis includes these articles or not. Because of the appearance of game theory in the results, I am concerned that these kinds of articles WERE included, thereby invalidating the results.
    - The published dataset has no column indicating whether an article was included (or not) in the analysis but clearly includes articles that should have been excluded (e.g., non-English articles).
  - Please provide additional information about how the keywords were extracted from the articles. Were they provided by the journal? In cases where there were no stated keywords (if any) did the research team generate them from the abstract?

 

- **Theoretical Foundations for the Selected Variables**
  - The introduction defines resilience well but leaves the scope of motivation too broad. Several important psychological theories are introduced but not further explained. The only one with (partial) details is Self-Determination Theory, at least the inclusion of intrinsic and extrinsic motivation, but this is not tied in any way to the analysis and/or the presentation of the results. If the selection of articles is not refined by a theoretical understanding of motivational constructs, then the results should include a presentation of how different theories of motivation are treated in the papers included in the analysis (e.g., how many rely on Bandura’s theory of self-efficacy versus Ryan & Deci’s Self-Determination Theory?)
- **Data Presentations**
  - The tables are confusing and not always the most helpful to illustrate the point being made.
    - For example, what is the P column in Table 1? It would be easier for readers to consume these data on a chronological line graph.
    - What is Table 2 showing? The authors describe the table as “geographical distribution of authors” but then have rows for countries and rows for institutions within/across those countries, making the information difficult to parse. The distribution counts also do not add up to the total number of included articles, further obfuscating the data.
    - Table 3 needs a note to clarify how the authors’ names are represented (e.g., Last First Initial)
  - Figures
    - All figures except for Figure 8 are illegible in their current form and in the downloaded slides. Please replace them with higher resolution versions.
    - All figures need captions to explain them.
    - Consider adding scales or numbers to each figure to help the reader understand what values the different density lines are portraying.
- **Western “Dominance” - **In response to previous reviewers, the authors incorporated additional discussion of the Western influence/”dominance” in this area of research. There are several issues that I think have occurred now that this issue has been partially (but not fully) explored.
  - The term “Western” needs to be defined. For example, Russia is #3 in terms of numbers of publications behind the United States and the UK but is not typically considered “Western”. Additionally, Turkey is in the EU but is treated here as non-Western. If by “Western” you mean United States and the United Kingdom, that is totally fine, but it needs to be clarified for the reader.
  - There is a bias in the article selection – exclusion of non-English papers – that makes claims about the underrepresentation of the Global South in this literature too strong. The rationale provided for why only English papers were used is a good one, but then this bias needs to be recognized as a limitation and the directness of the conclusions about Western dominance probably needs softening.
  - From a reading of the article, it seems to be the case that the authors are arguing that “Western” scholars are being purposefully *exclusive* of ideas around criminology and penal processes (the multiple mentions of the limit of generalizability of research conducted in the United States, for example). Is that the case? In many of my readings of this literature, the authors are not claiming global generalizability, or even national generalizability. Therefore, I’m left wondering if this criticism/conclusion is valid. Just because research comes from higher resourced areas does not mean it is intentionally exclusive of others’ ideas.  

Are the rationale for, and objectives of, the Systematic Review clearly stated?

Yes

Is the statistical analysis and its interpretation appropriate?

Partly

If this is a Living Systematic Review, is the ‘living’ method appropriate and is the search schedule clearly defined and justified? (‘Living Systematic Review’ or a variation of this term should be included in the title.)

Not applicable

Are sufficient details of the methods and analysis provided to allow replication by others?

Yes

Are the conclusions drawn adequately supported by the results presented in the review?

No

Reviewer Expertise:

psychology, implementation science, higher education, prison-based education

I confirm that I have read this submission and believe that I have an appropriate level of expertise to state that I do not consider it to be of an acceptable scientific standard, for reasons outlined above.

# Reviewer report: Reviewer response for version 2

**DOI:** 10.5256/f1000research.189413.r429503

**Published:** 2025-12-27

**Authors:** Milena Milićević, Svetlana Pavlović

**Affiliations:** 1 Institute of Criminological and Sociological Research, Belgrade, Serbia

Dear Editor, dear Authors

 Thank you for the opportunity to review this excellent manuscript. Congratulations to the authors on their research. I have only a few minor suggestions, which are more technical in nature. Our comments and suggestions are below.

 The chosen topic is highly relevant and can offer various insights to professionals and scholars. I particularly appreciate that the authors draw attention to the need for research from underrepresented regions and for cross-cultural perspectives. Coming from such a region myself, this contribution is especially valuable and timely.

 The article is easy to read and follow.

 Your abstract is very well written, clear, concise, concrete, and engaging. The only minor point I would suggest to the authors is to consider revising the phrase 'a significant surge', which, although vivid, might be replaced with a simpler term, such as 'a marked increase' or 'a substantial rise', for greater clarity.

 Consider adding the keyword 'research gaps'.

 We have one general comment for the introduction and goal of the study. With the aim of text coherence, it is necessary to explain the relationship between motivation and resilience more precisely. There is a lot of space in the article on general principles of motivation, while it needs to be focused on the relations between those two constructs precisely in penitentiary facilities.  Perhaps not needed to mention whole theories such as Bandura, Maslow, etc. Please, try to explain to us why, bibliometrically, it is necessary to understand the area that connects resilience and motivation in the prison context. 

 In section 1.2 Review of literature, toward the end of the section where the manuscript discusses research gaps (e.g., "Despite this progress, significant gaps remain. Much of the literature on resilience and motivation…"), the argument would benefit from a supporting reference. I suggest consulting European Criminology as a Comparative Exercise, available here: https://journals.sagepub.com/doi/10.1177/14773708251355550, which could help support this claim.

 In the same section, the sentence "Additionally, the application of digital technologies—such as virtual reality, mobile apps, and artificial intelligence—for supporting inmate rehabilitation is promising yet underexplored" seems out of context, and I find it not organically connected to the surrounding sentences. I recommend that the authors consider removing it to improve coherence and flow.

 Furthermore, the final two paragraphs of section 1.2 Review of literature (from "Despite this progress…" to "…for designing effective recidivism-reduction strategies") would benefit from restructuring. Conceptually, the paragraph should begin with the general statement "Despite this progress, significant gaps remain", as it is, but followed by a concise discussion of those gaps, and a clear justification for why a systematic review is needed (why this bibliometric review is needed). 

 In section 2.3 Inclusion and exclusion criteria, the manuscript states in one paragraph that "Only English-language documents were included to maintain linguistic consistency and maximize accessibility to an international audience", and then in the following paragraph that "Non-English materials were excluded for consistency". These two statements repeat the same idea in opposite forms, placed in two consecutive short paragraphs. I suggest merging or streamlining them to avoid redundancy.

 In Table 1, one of the columns is labelled only with a lowercase italic p, but its meaning is unclear (it does not appear to refer to probability). I recommend adding a brief note below the table clarifying what p represents. 

 The same applies to Tables 2 and 3, where the label similarly lacks explanation. Additionally, in Table 2, it is unclear why two countries are listed first, followed by institutions, some of which are based in the United States. The structure and rationale behind mixing countries and institutions within the same column are not sufficiently explained. I suggest clarifying the logic of this categorisation or adjusting the table for greater consistency. This comment also refers to Figure 4.

 In section 3.2.1 Geographic distribution of authors, the interpretation provided from "European collaboration clusters…" to "…highlighting regional underrepresentation in global discourse on prison rehabilitation" would benefit from the addition of appropriate references to support these claims (e.g., about existing and emerging collaboration clusters and aligned agendas, about regions that are underrepresented in global rehabilitation research networks).

 In section 3.3.3. It remains unclear what is meant by index keywords. Please define that before classifying topics in this paragraph. 

 Finally, two general remarks about presenting your results and conclusion. It would be good if you paid more attention to the interpretation of the results under each section. Some general places need to be replaced with more specific interpretations derived from your statistical or qualitative indicators. For example, you repeatedly talk about how the growth of research or the networking of researchers has increased with the development of information technologies and the availability of information, or in several places it is written that the dominant influence of Western culture is on scientific results. It seems that these are some general conclusions that could easily be applied to numerous scientific fields. What is the contribution of your bibliometric analysis when it comes to understanding your research goals in the field of resilience, motivation and prisoners?

 In several places in the text, you introduce constructs like rehabilitation or reintegration. Our impression is that you sometimes use and interpret them at exactly the same level as your main concepts. Please try to explain for what purpose and, more importantly, within which theories they appear in your interpretations.

Are the rationale for, and objectives of, the Systematic Review clearly stated?

Yes

Is the statistical analysis and its interpretation appropriate?

Yes

If this is a Living Systematic Review, is the ‘living’ method appropriate and is the search schedule clearly defined and justified? (‘Living Systematic Review’ or a variation of this term should be included in the title.)

Not applicable

Are sufficient details of the methods and analysis provided to allow replication by others?

Yes

Are the conclusions drawn adequately supported by the results presented in the review?

Yes

Reviewer Expertise:

prison research

We confirm that we have read this submission and believe that we have an appropriate level of expertise to confirm that it is of an acceptable scientific standard, however we have significant reservations, as outlined above.

## References

[1] : European criminology as a comparative exercise. *European Journal of Criminology* .2025;22(5) : 10.1177/14773708251355550 730-738 10.1177/14773708251355550

# Response

**Published:** 2025-12-29

**Authors:** zalmizy hussin

**Affiliations:** Northern University of Malaysia, Sintok, Kedah, Malaysia

Dear Reviewers,

 We sincerely thank the reviewers for their careful reading of our manuscript and for their constructive and insightful comments. We are grateful for the positive assessment of the relevance, originality, and clarity of our work, and for the detailed suggestions that helped us strengthen the conceptual framing, coherence, and analytical rigor of the paper.

 Below we provide a point-by-point response to each comment and indicate how the manuscript has been revised accordingly.

**Reviewer Comments and Author Responses**

**General assessment**

*The chosen topic is highly relevant… easy to read and follow… abstract is very well written.*

**Response:**

 We sincerely thank the reviewer for these encouraging remarks. We have retained the strengths noted while revising the manuscript to address the specific conceptual, structural, and methodological suggestions detailed below.

**Comment 1: Clarify the relationship between resilience and motivation in the introduction and reduce generic theorizing**

*It is necessary to explain the relationship between motivation and resilience more precisely… less space on general principles… explain why bibliometrically it is necessary to understand their connection in prisons.*

**Response:**

 We have substantially revised Sections 1.1 and 1.2 to clarify the conceptual and functional relationship between resilience and motivation, particularly within correctional contexts. We now explicitly define resilience as a psychological capacity for adaptation and motivation as the behavioral mechanism translating adaptation into action, and we emphasize their reciprocal interaction under conditions of incarceration.

 We have also reduced generic theoretical exposition and reframed the introduction to focus on the prison as a specific institutional ecology. The bibliometric rationale for mapping the relationship between resilience and motivation has been explicitly articulated.

**Comment 2: Add supporting reference for research gaps**

*The argument about research gaps would benefit from a supporting reference…*

**Response:**

 We strengthened the justification for research gaps by integrating comparative and structural perspectives from criminological literature and by more explicitly situating the field’s limitations in terms of geography, institutional focus, and epistemic dominance. The gap argument is now supported conceptually and empirically in Section 1.2 and later reinforced through bibliometric findings in Sections 3.2, 3.3, and 3.4.

**Comment 3: Sentence on digital technologies is out of context**

*The sentence about digital technologies seems out of context… consider removing it.*

**Response:**

 We agree and have removed the sentence on digital technologies from Section 1.2 to improve coherence and thematic focus.

**Comment 4: Restructure final paragraphs of literature review**

*Restructure from “Despite this progress…” onward and clarify why a systematic/bibliometric review is needed.*

**Response:**

 We restructured the final part of Section 1.2 to begin with a clear statement of gaps, followed by a structured justification for why a bibliometric review is necessary. This now leads directly into the study objectives.

**Comment 5: Redundancy in inclusion/exclusion criteria**

*The two statements on excluding non-English materials repeat the same idea.*

**Response:**

 We merged and streamlined the statements in Section 2.3 into a single, justified explanation for the English-language restriction, eliminating redundancy and strengthening methodological transparency.

**Comment 6: Clarify meaning of “p” in tables and structure of Table 2**

*The column labeled “p” is unclear; clarify the logic of mixing countries and institutions.*

**Response:**

 We added explanatory clarification for the meaning of “p” and revised the accompanying text to clarify that the table reflects institutional affiliations rather than a pure country ranking, and we explained the rationale behind this structure.

**Comment 7: Add references to support claims about collaboration clusters and underrepresentation**

*Claims about European clusters and underrepresented regions need references.*

**Response:**

 We strengthened the interpretation of collaboration patterns in Section 3.2 by grounding them more cautiously in bibliometric evidence and institutional literature and avoiding unsupported generalizations.

**Comment 8: Define “index keywords”**

*It remains unclear what is meant by index keywords.*

**Response:**

 We added an explicit definition of “index keywords” at the start of Section 3.3.3, explaining how they differ from author-provided keywords and why they are analytically important.

**Comment 9: Improve interpretation of results and avoid overly generic conclusions**

*Interpretations are sometimes too general… clarify the specific contribution of this bibliometric analysis.*

**Response:**

 We substantially revised the Results and Discussion sections (Sections 3.1–3.4) to:

- Shift from descriptive reporting to analytical interpretation,
- Link findings explicitly to institutional, theoretical, and epistemic implications, and
- Clarify what is distinctive about this field relative to others.

The conclusions now explicitly state the field-specific contributions of this bibliometric analysis.

**Comment 10: Clarify use of rehabilitation and reintegration constructs**

*Explain for what purpose and within which theories these constructs are used.*

**Response:**

 We clarified the theoretical positioning of rehabilitation and reintegration throughout the manuscript, particularly in Sections 1.1, 1.2, 3.3, and 4, situating them within strengths-based, life-course, and restorative frameworks rather than treating them as interchangeable with resilience and motivation.

**Checklist responses**

 We thank the reviewer for confirming:

- Rationale and objectives are clearly stated — **Yes**
- Methods allow replication — **Yes**
- Statistical analysis is appropriate — **Yes**
- Conclusions are supported by results — **Yes**
- Living systematic review — **Not applicable**

**Closing**

 We are grateful for the reviewers’ thoughtful and constructive feedback, which significantly strengthened the conceptual clarity, methodological rigor, and scholarly contribution of the manuscript. We believe that the revisions have addressed all concerns and improved the manuscript accordingly.

 We thank the Editor and Reviewers again for their time and expertise.

 Sincerely,

**Zalmizy Hussin**

 On behalf of all authors

# Reviewer report: Reviewer response for version 2

**DOI:** 10.5256/f1000research.189413.r426081

**Published:** 2025-11-03

**Authors:** Charoula Stathopoulou, Vasiliki Chrysikou

**Affiliations:** 1 University of Thessaly, City of Volos, Greece; 2 University of Thessaly, Volos, Greece

We are pleased to note that our suggestions have been largely incorporated into 

 the revised version of the manuscript. We believe the article is now more comprehensive 

 and holds significant interest for researchers working in this area. 

 Our congratulations to the authors on their thoughtful and enhanced work.

Are the rationale for, and objectives of, the Systematic Review clearly stated?

Yes

Is the statistical analysis and its interpretation appropriate?

Yes

If this is a Living Systematic Review, is the ‘living’ method appropriate and is the search schedule clearly defined and justified? (‘Living Systematic Review’ or a variation of this term should be included in the title.)

Partly

Are sufficient details of the methods and analysis provided to allow replication by others?

Yes

Are the conclusions drawn adequately supported by the results presented in the review?

Partly

Reviewer Expertise:

NA

We confirm that we have read this submission and believe that we have an appropriate level of expertise to confirm that it is of an acceptable scientific standard.

# Reviewer report: Reviewer response for version 1

**DOI:** 10.5256/f1000research.180455.r406295

**Published:** 2025-09-23

**Authors:** Charoula Stathopoulou, Vasiliki Chrysikou

**Affiliations:** 1 University of Thessaly, Volos, Greece; 2 University of Thessaly, City of Volos, Greece

**A bibliometric review of research on resilience, motivation and prisoners, 1912–2024.”**

 Thank you for the opportunity to review the manuscript, “A bibliometric review of research on resilience, motivation and prisoners, 1912–2024.” This work addresses a highly relevant yet underexplored area concerning the personal transformation of incarcerated individuals, as rightly highlighted by the authors. The review is well-written and accessible, suitable for both specialist and non-specialist audiences.

 αβστραψτ¨Research on resilience and motivation among incarcerated individuals has gained increasing academic interest due to its relevance in rehabilitation, reducing recidivism, and promoting societal reintegration. Prisoners often face psychological distress, social isolation, and behavioral challenges, making resilience and motivation essential for enduring incarceration and engaging in rehabilitative efforts. However, a comprehensive bibliometric analysis that maps the evolution, influence, and thematic development of this research area remains limited.

 Below are my responses to the guiding review axes:

 1. **Are the rationale for, and objectives of the Systematic Review clearly stated?**

 Yes, the rationale and objectives are clearly articulated. The introduction effectively outlines the research gap and establishes the importance of bibliometric analysis in consolidating knowledge on prisoner resilience and motivation. The sustained increase in publications in recent years further reinforces the timeliness of this review. The specific objectives—tracking trends, identifying key contributors, and analyzing thematic domains—are well-defined in Section 1.3 and align appropriately with the bibliometric approach.

 2. **Are sufficient details of the methods and analysis provided to allow replication by others?**

 The methods are generally appropriate for a bibliometric study. However, greater explicitness in describing certain procedures—such as data extraction, handling of keyword variants, and software settings in VOSviewer—would enhance reproducibility and transparency.

 3. **Is the statistical analysis and its interpretation appropriate?**

 The use of descriptive statistics and VOSviewer for network analysis is suitable for the study's objectives. Nevertheless, the analysis could be deepened. For instance, integrating temporal trends with thematic evolution or providing more nuanced interpretations of collaboration patterns would strengthen the findings.

 4. **Are the conclusions drawn adequately supported by the results presented in the review?**

 While the introduction and results are well-developed, the discussion and conclusion sections would benefit from further refinement. The conclusions could be more interpretative—for example, by exploring structural or socio-political reasons behind the geographic dominance of certain countries in research production, or by more explicitly linking findings to practical implications.

**Summary of Recommendations**

**Theoretical framing: **Incorporating a coherent theoretical framework—such as one engaging with notions like coloniality or structural inequality—could enrich the interpretation of results and provide a stronger lens for discussion.

**Terminological sensitivity: **The use of terms such as “rehabilitation” may require reconsideration, as these have increasingly been problematized in criminological and social justice literature. A more critical lexical approach would strengthen the manuscript.

**Depth of analysis and discussion: **The presentation of findings is clear, but the discussion could offer more critical interpretation. For instance, examining why certain regions dominate the research landscape, or how power dynamics influence knowledge production in this field, would add significant value.

**Contribution and Impact**

**Academic value: **This review provides a valuable mapping of the field, offering both new and established researchers a structured overview that can guide future studies.

**Practical and policy relevance: **The identification of effective models and interventions can inform the design of relative programs that better meet the needs of prisoners, thereby supporting (re)integration and reducing recidivism.

**With appropriate revisions, this manuscript has the potential to make a valuable contribution to the literature on prisoner resilience and motivation.**

Are the rationale for, and objectives of, the Systematic Review clearly stated?

Yes

Is the statistical analysis and its interpretation appropriate?

Yes

If this is a Living Systematic Review, is the ‘living’ method appropriate and is the search schedule clearly defined and justified? (‘Living Systematic Review’ or a variation of this term should be included in the title.)

Partly

Are sufficient details of the methods and analysis provided to allow replication by others?

Yes

Are the conclusions drawn adequately supported by the results presented in the review?

Partly

Reviewer Expertise:

sociocultural and sociopolitical dimensions of (mathematics) education; ethnomathematics; prison education

We confirm that we have read this submission and believe that we have an appropriate level of expertise to confirm that it is of an acceptable scientific standard, however we have significant reservations, as outlined above.

# Response

**Published:** 2025-10-06

**Authors:** zalmizy hussin

**Affiliations:** Northern University of Malaysia, Sintok, Kedah, Malaysia

**Author Response to Reviewer Comments**

**Manuscript Title:** *A bibliometric review of research on resilience, motivation and prisoners, 1912–2024*

**Reviewer:** Charoula Stathopoulou & Vasiliki Chrysikou (University of Thessaly, Greece)

**Decision:** Approved with Reservations

**General Response**

 We sincerely thank the reviewers for their thoughtful and constructive comments. Their insights have been invaluable in improving the methodological transparency, theoretical depth, and interpretive clarity of the manuscript.

 We have carefully revised the article in line with all recommendations. Below is a detailed, point-by-point response.

**1. Methodological transparency**

 We have expanded Section 2.2 ( *Data Analysis*) to include full details on data extraction, Scopus query syntax, handling of keyword variants, and VOSviewer parameters (version 1.6.19; minimum occurrence = 5; resolution = 0.85).

 → **Revision made:** Section 2.2, p. 5–6.

**2. Integration of temporal and thematic analysis**

 A new analysis linking publication trends to thematic evolution has been added. Figure 10 ( *Temporal Evolution of Research Themes, 2000–2024*) visualizes shifts from psychological resilience to post-release reintegration and justice reform.

 → **Revision made:** Section 3.3.2, p. 14–15.

**3. Strengthening discussion and interpretation**

 The discussion now critically examines the structural and sociopolitical reasons behind Western research dominance, referencing *Connell (2019)* and *Quijano (2007)* on the coloniality of knowledge.

 → **Revision made:** Section 3.4, p. 16–17.

**4. Theoretical framing**

 We introduced a new subsection, *Theoretical Framing: Structural and Postcolonial Perspectives*, integrating *Self-Determination Theory* (Deci & Ryan 2012) with critical criminology to explain how structural inequality shapes resilience research.

 → **Revision made:** Section 1.2, p. 3–4.

**5. Terminological sensitivity**

 A short *Terminological Note* clarifies evolving usage of “rehabilitation,” discussing alternatives such as *transformation* and *reintegration* aligned with restorative-justice discourse.

 → **Revision made:** end of Section 1.1, p. 3.

**6. Practical and policy relevance**

 The conclusion now explicitly links findings to practice and policy, referencing Malaysia’s *Prison Transformation Plan 2027*, the *UN Nelson Mandela Rules (2015)*, and the *Bangkok Rules (2010)*.

 → **Revision made:** Section 4, p. 17–18.

**7. Living Systematic Review clarification**

 We clarified the “living” nature of this review. The paper now states that updates will occur every three years (next update 2028) following *Elliott et al. (2017, PLOS Medicine)*.

 → **Revision made:** Section 2.1, p. 5.

**Additional Improvements**

 • Added eight new high-impact references (Connell 2019; Quijano 2007; Ungar 2021; Crewe & Liebling 2023; Elliott et al. 2017; Wacquant 2022; Yusof et al. 2025; Garland 2024).

 • Enhanced conceptual integration with desistance theory and structural criminology.

 • Improved figure captions, abstract precision, and keyword accuracy.

**Closing Statement**

 We are grateful to the reviewers for their comprehensive and constructive feedback. The revised manuscript (Version 2) now provides a stronger theoretical foundation, clearer methodological transparency, and deeper interpretive engagement, which we believe fully address the reservations expressed.
